# Molecular and Functional Evolution of the Spermatophyte Sesquiterpene Synthases

**DOI:** 10.3390/ijms22126348

**Published:** 2021-06-14

**Authors:** Dongmei Liang, Weiguo Li, Xiaoguang Yan, Qinggele Caiyin, Guangrong Zhao, Jianjun Qiao

**Affiliations:** 1 Department of Pharmaceutical Engineering, School of Chemical Engineering and Technology, Tianjin University, Tianjin 300072, China; ldmxp@tju.edu.cn (D.L.); liweiguo@tju.edu.cn (W.L.); yanxiaoguang@tju.edu.cn (X.Y.); qinggele@tju.edu.cn (Q.C.); grzhao@tju.edu.cn (G.Z.); 2 Key Laboratory of Systems Bioengineering, Tianjin University, Ministry of Education, Tianjin 300072, China; 3 SynBio Research Platform, Collaborative Innovation Center of Chemical Science and Engineering (Tianjin), Tianjin 300072, China; 4 Frontiers Science Center for Synthetic Biology, Tianjin University, Ministry of Education, Tianjin 300072, China

**Keywords:** spermatophyte sesquiterpene synthetase, sesquiterpene, phylogenetic analysis, functional evolution

## Abstract

Sesquiterpenes are important defense and signal molecules for plants to adapt to the environment, cope with stress, and communicate with the outside world, and their evolutionary history is closely related to physiological functions. In this study, the information of plant sesquiterpene synthases (STSs) with identified functions were collected and sorted to form a dataset containing about 500 members. The phylogeny of spermatophyte functional STSs was constructed based on the structural comparative analysis to reveal the sequence–structure–function relationships. We propose the evolutionary history of plant sesquiterpene skeletons, from chain structure to small rings, followed by large rings for the first time and put forward a more detailed function-driven hypothesis. Then, the evolutionary origins and history of spermatophyte STSs are also discussed. In addition, three newly identified STSs *CaSTS2*, *CaSTS3,* and *CaSTS4* were analyzed in this functional evolutionary system, and their germacrene D products were consistent with the functional prediction. This demonstrates an application of the structure-based phylogeny in predicting STS function. This work will help us to understand evolutionary patterns and dynamics of plant sesquiterpenes and STSs and screen or design STSs with specific product profiles as functional elements for synthetic biology application.

## 1. Introduction

Terpenes constitute a large class of chemically and structurally diverse natural products in the plant kingdom and serve multiple physiological and ecological functions. At present, more than 25,000 terpenoid structures and 80,000 compounds have been found [1]. Sesquiterpenes are the most complex group with structural diversity, and more than 300 kinds of basic skeletons have been found, which are widely distributed in plants and microorganisms [2]. Sesquiterpenes play important roles in interactions with pollinators and seed dispersers [3], direct defenses against herbivores [4] and pathogens [5], mediate plant–plant and plant–microbe interactions [6], and help acclimation to biotic and abiotic environmental stress [7].

**Figure 1:**
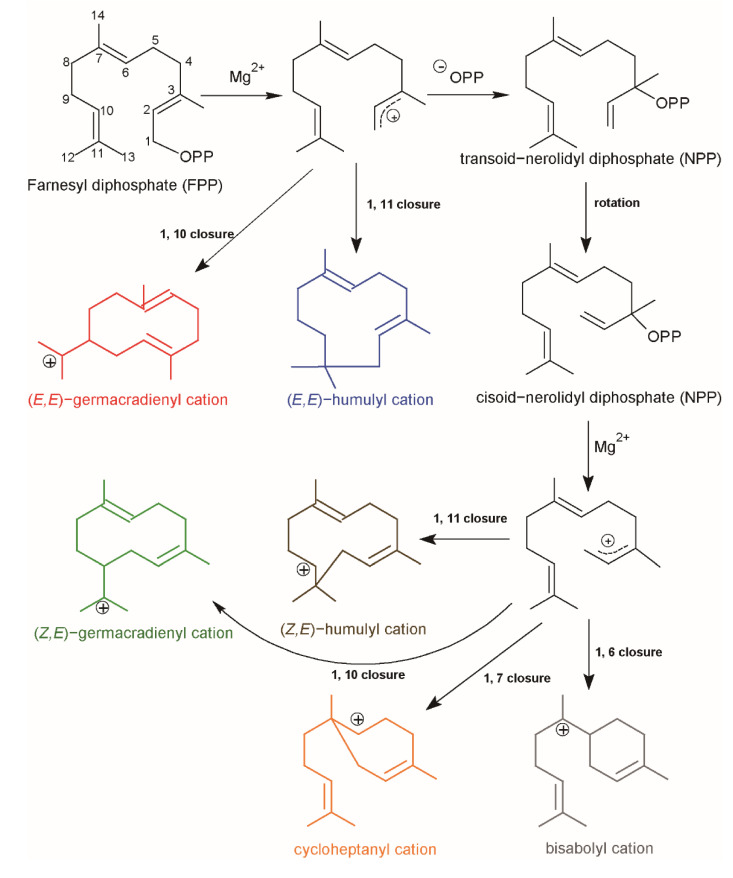
The reaction mechanism of sesquiterpene production starting with FPP. Farnesyl cation is formatted by losing the diphosphate moiety (OPP). Then, the farnesyl cation can be converted to the nerolidyl cation. Possible cyclizations for both cations are indicated in the figure.

The main structure of sesquiterpene is biosynthesized by STSs, which convert farnesyl diphosphate (FPP) into the sesquiterpene skeleton [8]. Common to all STSs is the formation of highly reactive carbocationic intermediates which can undergo a great variety of rearrangements resulting in a huge number of different sesquiterpene structures [9]. It is initiated by the divalent metal ion-dependent ionization of the substrate FPP, and the following cyclization reactions depending on which carbon−carbon double bond reacts with the initially formed allylic carbocation (Figure 1) [10]. One type involves cyclization of farnesyl cation to yield (*E,E*)-germacradienyl cation (C10-C1 closure) or (*E*)-humulyl cation (C11-C1 closure) rings. The other type is initiated by isomerization of the C2–C3 double bond of farnesyl cation to the tertiary nerolidyl cation. Then, the cisoid conformer of nerolidyl cation can undergo cyclization to either the central or distal double bond forming bisabolyl cation (C6-C1 closure), cycloheptanyl cation (C7-C1 closure), (Z,E)-germacradienyl cation (C10-C1 closure), or (Z)-humulyl cation (C11-C1 closure). The resulting cationic intermediate undergoes deprotonation or addition of water before termination sesquiterpene [1].

The ancestral bifunctional diterpene synthases having γβα tri-domain architecture, presumably catalyzing production of gibberellin intermediate ent-kaurene, seem to the ancestor of all plant terpene synthases (TPS) [11]. The ancient gene duplication and sub-functionalization led to separate class II diterpene cyclases (γβα tri-domain) and subsequently class I TPSs (βα didomain) including the STSs [12]. At present, most studies on STS evolution focused on the phylogenetic location of STSs [13,14]. The TPS family is classified into seven subfamilies designated as TPS-a, TPS-b, TPS-c, TPS-d, TPS-e/f, TPS-g, and TPS-h [11]. Among these, the STSs are predominantly distributed in the angiosperm-specific TPS-a clade and the gymnosperm-specific TPS-d clade [12], and there are few STSs in the TPS-b, TPS-g, and TPS-e/f subfamilies [11]. However, functional evolution of plant STSs has not yet been clarified. It is difficult to predict enzyme function from STS sequences, because STSs represent a very diverse set of enzymes with a wide range of sequence similarities. Janani et al. gathered 262 plant STS sequences with experimentally characterized products, hoping to choose likely functional residues for mutagenesis studies; unfortunately, they did not reveal any general rules for STS function prediction [15]. 

In this study, we collected a dataset of spermatophyte STSs with characterized products from public databases and the literature. In total, 394 spermatophyte STSs were analyzed to explore the functional formation and evolution of plant sesquiterpenes. Based on structure-guiding phylogeny, we investigated the phylogenetic relationships and product patterns in each clade and speculated the possible evolutionary model of spermatophyte STSs. The association analysis of STS evolutionary history and product patterns revealed the inherent correlation between STS sequences and their functions, providing the possibilities for function prediction of STSs. In addition, three newly identified STSs were analyzed based on the STS functional evolution pattern to assess the instructional significance on STS functional prediction. Finally, we speculated the evolutionary origin and history of spermatophyte STSs and put forward a more detailed function-driven hypothesis. Overall, this study provides new insights into the possible evolutionary scenario for spermatophyte sesquiterpenes and STSs.

## 2. Results

### 2.1. Dataset of Characterized Spermatophyte STSs

**Figure 2:**
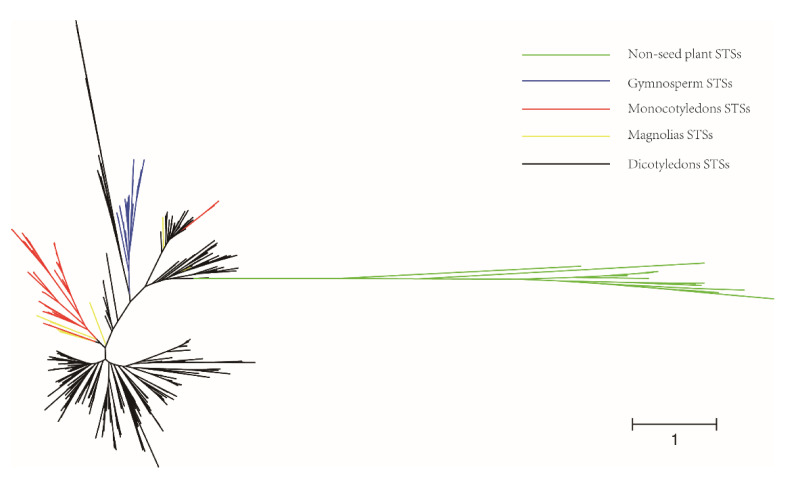
Phylogeny of functional plant STSs. Green clades represent non-seed plant STSs, blue clades represent gymnosperm STSs, red clades represent monocotyledon STSs, yellow clades represent magnolias STSs, and black clades represent dicotyledon STSs.

To obtain a comprehensive set of functional STSs, we searched for the public databases, a subset of UniProt, NCBI, and Database of Characterized Plant Sesquiterpene Synthases (http://www.bioinformatics.nl/sesquiterpene/synthasedb/) in 10 January 2021, and manually reviewed literature linked to enzymes with the characteristic STS domain. Overall, we present a dataset of 474 manually curated characterized plant STSs. Among these, 421 STSs have sequence information, including 27 STSs from non-seed plants, 29 STSs from gymnosperm, and 365 STSs from angiosperm. The information of the 474 STSs, including gene name, species origin, GenBank, products (major and minor), and product type, is presented in Tables S1–S3. The dataset supports searching and sorting of all or subsets of the data. To investigate the functional evolution of plant STSs, our dataset covered not only typical plant STSs, but also monoterpene and diterpene synthases with STS ability. The phylogenetic tree of all 421 plant STSs (Figure 2) showed that non-seed plant STSs (green) clustered obviously, and they were relatively independent from other plant STSs. The gymnosperm STSs (blue) also clustered in a single clade, which showed the evolutionary difference between them and angiosperm STSs. 

**Figure 3:**
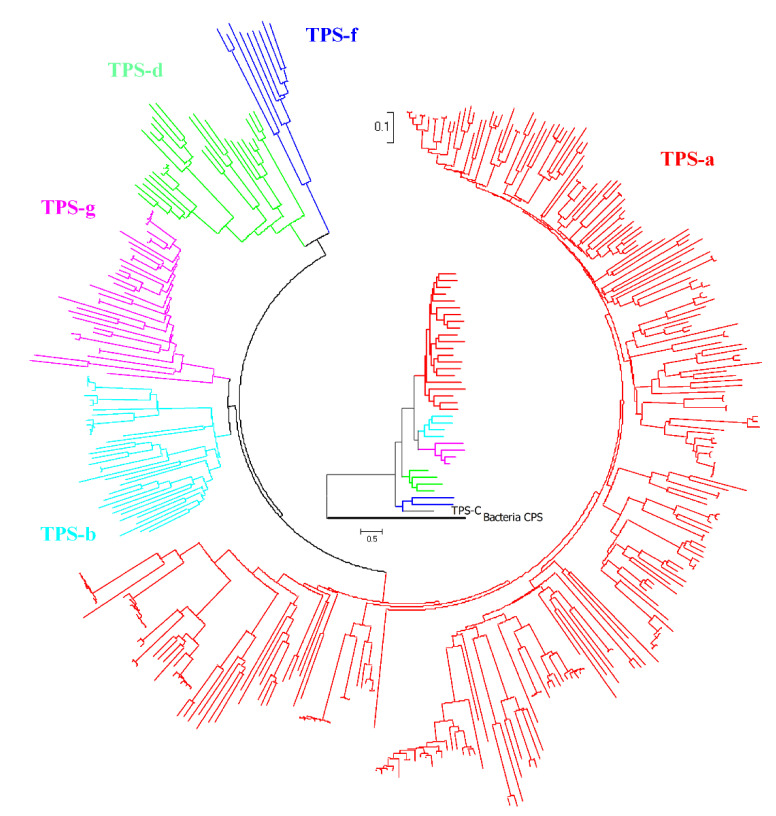
Phylogeny of functional spermatophyte STSs. Outer ring is the phylogenetic tree of all 394 spermatophyte STSs, inner ring is the global phylogenetic tree of representative plant STSs. Red branches represent TPS-a subfamily STSs, light blue branches represent TPS-b subfamily STSs, green branches represent TPS-d subfamily STSs, blue branches represent TPS-f subfamily STSs, pink branches represent TPS-g subfamily STSs.

We manually curated 447 characterized spermatophyte plant STSs, among which 394 STSs have sequence information (Tables S2 and S3). This is the largest and most diverse group of STSs currently known. The structure-based phylogenetic tree of all 394 spermatophyte STSs (Figure 3) showed that most angiosperm STSs belong to the TPS-a subfamily (red), while gymnosperm STSs belong to the TPS-d subfamily (green). In addition, a few STSs can be summarized as TPS-f (blue), TPS-g (pink), and TPS-b (light blue) subfamilies (Figure 3 outer ring). In order to show the evolutionary position of STSs in each clade more clearly, we selected 35 representative STSs (shaded in Tables S2 and S3) based on the clade location, species source, and product type to construct global phylogenetic tree (Figure 3, inner ring). The phylogeny of spermatophyte STSs is basically consistent with that of all plant TPSs [16]. The diterpene synthase clade (TPS-f) is the closest to the ancestral clade TPS-c, indicating that it might evolve from the ancestral bifunctional copalyl diphosphate synthase/*ent*-kaurene synthase (CPS/KS) [17]. Although gymnosperm STSs (the TPS-d clade) and angiosperm STSs derived from a common ancestor, they have an obvious evolutionary divergence, corresponding with the speculation that gymnosperms are monophyletic groups, which are far away from angiosperms [18]. The TPS-g clade contains many bifunctional monoterpene/sesquiterpene synthases, having many characteristics of the evolutionary transition state [16]. The neighbor clade TPS-b contains more actual monoterpene synthases, which indicates that most angiosperm STSs originated from a recent common ancestor and the ancestral protein gained the ability to produce sesquiterpenes through the continuous evolution from monoterpene synthases.

### 2.2. The Phylogeny of Spermatophyte STSs in the TPS-f Subfamily

**Figure 4:**
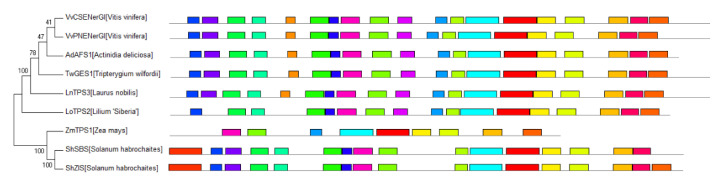
The phylogeny of spermatophyte STSs in the TPS-f subfamily. The colored boxes represent different conserved motifs in spermatophyte STSs predicted by MEME online tool (https://meme-suite.org/meme/) in 11 March 2021.

The TPS-f clade is closest to the common ancestor of typical plant TPSs, but the function and evolutionary process of the members are not the same. In order to better understand the STS evolutionary events in the TPS-f subfamily, we performed the phylogenetic analysis separately (Figure 4). Within the TPS-f subfamily, *LnTPS3* [19] and *TwGES1* [20] are real diterpene synthases and the main product is geranyllinalool. They also have the ability to produce acyclic sesquiterpene (*E*)-nerolidol with the substrate FPP. *VvCSENerG1* and *VvPNENerG1* from grape also show the same product spectrum [21]. *AdAFS1* have typical diterpene synthase conserved domain but can produce acyclic (*E,E*)-farnesene only [22]. The same product is also formed by *LoTPS2* lacking two conserved domains at the N-terminus [23]. 

In the other subclade of TPS-f, there are three actual STSs. There is a common sequence at the head of *ShSBS* and *ShZIS*, which is unique to TPS in tomato (Figure 4). Subcellular localization of *ShSBS* shows that it contains the plastid-targeting peptide and locates in the chloroplast. The first 36 residues of the common sequence may be responsible for chloroplast localization and play an important role in the evolution of these special TPSs. *ShSBS* and *ShZIS* may originate from the original TPS-e/f diterpene synthase, occasionally obtaining the ability to synthesize monoterpenes and sesquiterpenes through exon exchange [24]. *ZmTPS1* has almost completely lost the N-terminus protonation domain and is highly similar to maize KS, which is different from other TPS-f proteins that retain the basic structure of the original diterpene synthase, more similar to typical plant STSs [25]. *ZmTPS1* may have directly evolved from maize KS, but it has completely lost the ability to produce diterpenes. The phylogeny of STSs in the TPS-f subfamily indicates that TPS obtained the ability of producing sesquiterpene from the ancestral diterpene synthase by different evolutionary routes. 

### 2.3. The Phylogeny of Spermatophyte STSs in the TPS-d Subfamily

**Figure 5:**
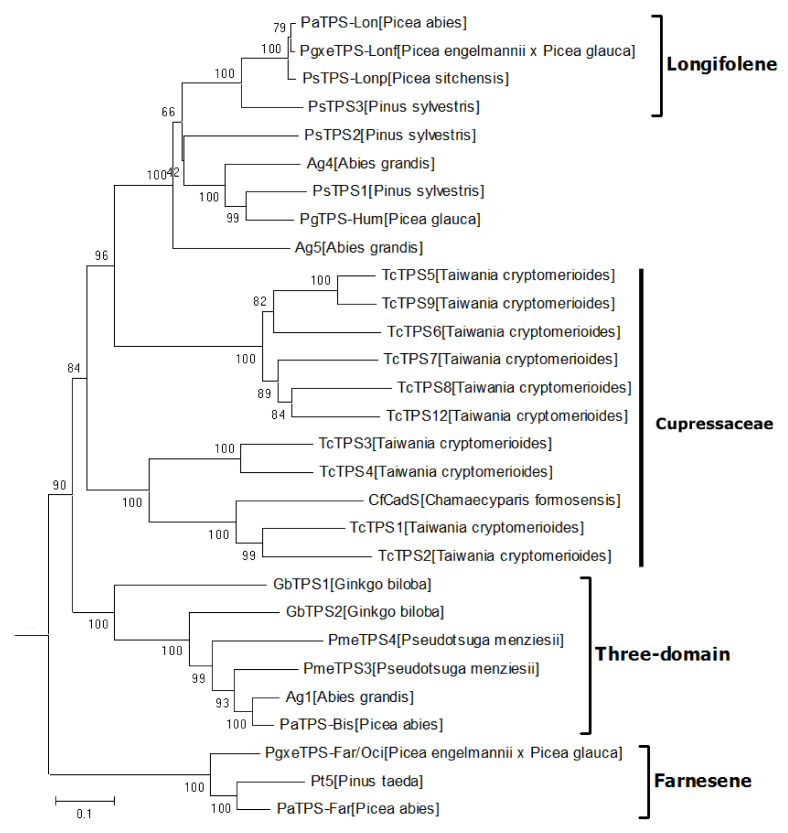
The phylogeny of spermatophyte STSs in the TPS-d subfamily.

All of the gymnosperm STSs phylogenetically clustered into the TPS-d subfamily [16], which are closer to the ancestral TPSs than angiosperm STSs. The clade TPS-d is comprised of 29 representative STSs from 12 different plant species (Figure 5), clustered generally according to the function. The outer part is the acyclic α-farnesene clade, which indicates that the earliest sesquiterpenes evolved in gymnosperms are probably acyclic. The three-domain clade contains STSs with γβα three domains, similar to ancestral bifunctional diterpene synthases, and their main product was bisabolene (C6-C1 closure), indicating that the ancestor of this clade was probably evolved from bisabolene synthases. The *TcTPSs* in the *Cupressaceae* group are divided into two subclades. *TcTPS1* and *TcTPS2* have nine introns that are the same as the published TPS-d members, while *TcTPS5-8* have lost intron XII [26]. The distant phylogenetic relationship and the intron loss event of *TcTPSs* correlate to the different product profile. *TcTPS1* and *TcTPS2* produce a major product zingiberene (C6-C1 closure), while other *TcTPSs* mainly produce sesquiterpenes formed by C10-C1 or C11-C1 closure. Interestingly, STSs in longifolene clade also form large-ring products (C11-C1 closure). Thus, we speculated that *TcTPS1-4* might be a transitional state of STS functional evolution from small ring (C6-C1 closure) to large ring (C10-C1 and C11-C1 closure). Overall, we proposed the possible evolutionary process of STSs in the TPS-d subfamily, that is, the chain products gradually evolved to form small rings and then to form large rings.

### 2.4. The Phylogeny of Spermatophyte STSs in the TPS-b/g Subfamily

**Figure 6:**
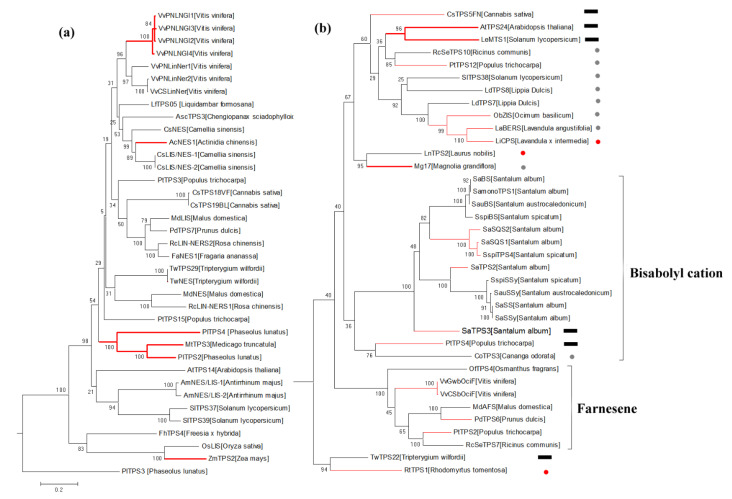
(**a**) The phylogeny of spermatophyte STSs in the TPS-g subfamily. STSs with diterpene synthases function in vitro are shown by red branches and uniformly distributed in the phylogenetic tree. (**b**) The phylogeny of spermatophyte STSs in the TPS-b subfamily. STSs with monoterpene synthases function in vitro are shown by red branches. STSs producing 1,6-cyclized sesquiterpenes and 1,10/1,11-cyclized sesquiterpenes are marked with grey dots and red dots, respectively. Products of STSs marked with black rectangles are acyclic farnesene or nerolidol.

Based on current knowledge, the TPS-b and TPS-g clades are the angiosperm-specific subfamilies [16]. They are closely related to the TPS-a clade and have substantially diverged from the other TPS clades (Figure 3). The TPS-g clade is comprised of 37 members producing acyclic products (Figure 6a), which are almost all bi- or tri-functional TPSs [21]. The STSs that can produce diterpenes in vitro (red branches in Figure 6a) are evenly placed in the TPS-g clade, indicating that the TPS-g ancestor might be a multifunctional diterpene synthase derived from TPS-f proteins. The TPS evolution involves not only a change in substrate specificity but also in subcellular localization. A relative recent example of such an event may exist in the *Antirrhnium majus* bifunctional TPS *AmNES/LIS-2* sharing 95% amino acid sequence identity with *AmNES/LIS-1*, which has an additional 30 amino acids in the N-terminus, leading to its localization in plastids and accounting for linalool formation, whereas *AmNES/LIS-1* is localized in cytosol and is responsible for nerolidol biosynthesis [27]. Another good example of neofunctionalization of duplicated STS genes involving a change in subcellular localization is the pair of *CsLIS/NES-1* and *CsLIS/NES-2* [28]. In contrast to the cytosolic STSs, most mono- and diterpene synthases have obvious N-terminus plastid transit peptides. The phylogeny of STSs in the TPS-g subfamily indicates that ancestral diterpene synthase gradually evolved to form monoterpene synthase and then to form acyclic STSs. The embryonic form of STSs was evolved by the expansion of the substrate spectrum. Then, STSs having a broad substrate spectrum undergo variable splicing or exon loss resulted in losing N-terminus plastid transit peptides, and gradually formed monofunctional STS under the restriction of FPP substrate pool. 

Three multifunctional TPSs of *PlTPS2*, *PlTPS3*, and *PlTPS4* from *Phaseolus lunatus* phylogenetically clustered into the TPS-g subfamily. They are likely derived from a duplication of an ancestral gene followed by subfunctionalization. *PlTPS3* can convert FPP to (E)-nerolidol, which is the precursor of the homoterpene (*E*)-4,8-dimethyl-1,3,7-nonatriene (DMNT) [29]. *PlTPS2* and *PlTPS4* can produce (*E,E*)-geranyllinalool from GGPP. Homoterpene (*E,E*)-4,8,12-trimethyl-1,3,7,11-tridecatetraene (TMTT) is then produced from (*E,E*)-geranyllinalool [29]. DMNT and TMTT are herbivore-induced plant volatiles, which enables the plants to attract natural enemies of herbivores [30]. Sesquiterpenes probably evolved gradually during the plants coping with insects and other environmental pressures. The phylogenetic position and physiological function of TPS-g STSs may suggest that (E)-nerolidol is the early form of plant sesquiterpenes. 

TPS-b (Figure 6b) is the sister group of TPS-g with more complicated evolutionary history, fortunately displays clustering characteristic of product structures. The outside Farnesene-branch contains 7 farnesene synthases from different sources, and most of the other STSs produce 1,6-cyclized sesquiterpenes (members in bisabolyl cation-branch and marked with grey dots). Three 1,10- or 1,11-cyclized STSs are sporadically distributed in the phylogenetic tree (red dot). Meanwhile, many TPS-b members have the activity of monoterpene synthase in vitro (red branches in Figure 6b), leading the TPS-b subfamily to present a transitional state of different product types of monoterpenes and sesquiterpenes. Therefore, we speculated that the possible evolutionary process of TPS-b members was similar to that of TPS-d members, that is, the chain products gradually evolved to form small rings (C6-C1 closure) and then to form large rings (C10-C1 and C11-C1 closure).

### 2.5. The Phylogeny of Spermatophyte STSs in the TPS-a Subfamily

**Figure 7:**
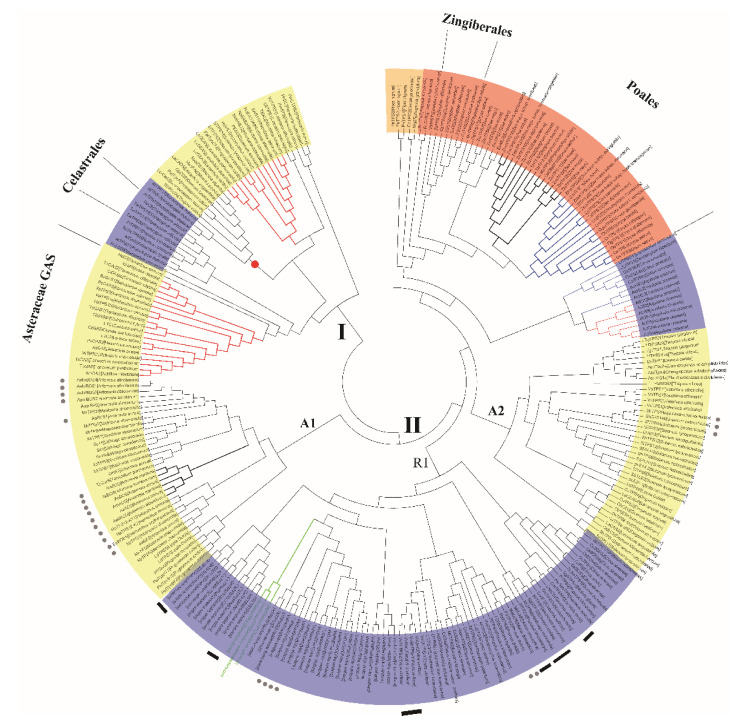
The phylogeny of spermatophyte STSs in the TPS-a subfamily. Blue shadow represents the *Rosanae* STSs, yellow shadow represents the *Asterids* STSs, red shadow represents the *monocot* STSs, and orange shadow represents the *magnoliid* STSs. Red branches show the STSs with identical products, blue branches show the STSs with 1,11-cyclized functions and green branches show the probable HGT members from *Santalum album*. STSs producing 1,6-cyclized sesquiterpenes are marked with grey dots, and the neighbor STSs producing 1,10- or 1,11-cyclized products, respectively, are marked with black rectangles.

TPS-a is the angiosperm-specific subfamily, which dominates the STS family in spermatophyte. The expansion of TPS-a subfamily may occur after the split of the monocot and dicot lineages by gene duplication and divergence, because of the obvious species-clustered phenomenon. The TPS-a clade is comprised of 282 representative STSs (Figure 7), in which monocotyledon and dicotyledon STSs clustered separately, indicating the common ancestor. In the monocot clade (red), STSs from *Zingiberales* and *Poales* are clustered separately (Figure 7), suggesting large-scale gene duplications which probably occurred not long ago. STSs are obviously functionally clustered in the *Poales* clade, including the 1,6-cyclized product branch (black) of representative bisabolene and 1,11-cyclized product branch (blue) of representative (E)-β-caryophyllene. All functionally characterized dicotyledon STSs in the TPS-a subfamily are from core eudicots, including *Rosanae* (blue) and *Asterids* (yellow). The state of clustered STSs in angiosperms is consistent with plant species classification, which indicates that STSs are developing with species evolution. 

TPS-a contains two major clades, among which, Clade II has more members: members in the A1 and A2 clades are all from *Asterales* and members in the R1 clade are mainly from *Rosanae*. In the A1 clade, STSs catalyzing initial 1,6-ring closures are mainly distributed in *Artemisia* (grey dot), and the other STSs mainly catalysis initial 1,10-ring closures. A few of 1,6-cyclized STSs (grey dots) are distributed in the A2 and R1 clades, and the others are 1,10/1,11-cyclized STSs, among which some neighbor STSs produce 1,10- or 1,11-cyclized products, respectively (marked with black rectangles), suggesting that conversion of these two structures are common in STS evolution. Although Clade I has fewer members, it contains all orders of *Asterids* and the most important fabids and malvids of the *Rosanae*. It can be considered that their ancestral proteins appeared early before the large-scale differentiation of dicotyledons. Interestingly, almost all germacrene A synthases (GASs) fell into this clade. Germacrene A is an intermediate catalyzed by several STSs to form eudesmane-type sesquiterpene [31], the characteristic products of *Asteraceae* and *Celastrates*. Germacrene A and (E)-β-farnesene are both aphid alarm pheromones and probable ancestral forms of plant sesquiterpenes, which naturally dovetails with the function-driven hypothesis. 

### 2.6. Predicting the STS Functions through Functional Evolution Analysis

**Figure 8:**
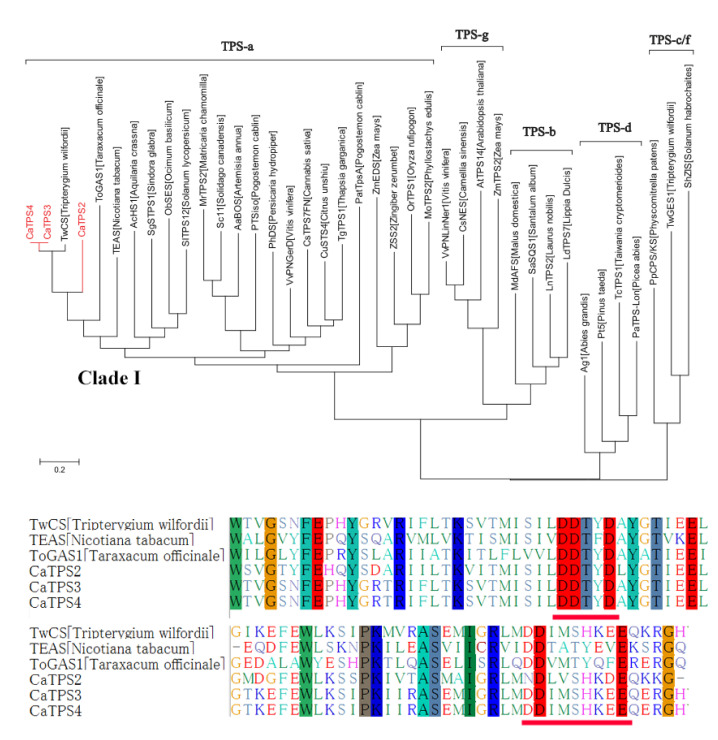
The phylogeny of candidate STSs in *C. angulatus*. Red branches represent the *CaTPSs*. The red underlines show the conserved DDxxD and NSE/DTE motifs.

Three STSs, *CaSTS2*, *CaSTS3,* and *CaSTS4,* were obtained from the *Celastrus angulatus* raw sequence reads deposited in NCBI (STUDY: PRJNA509518) [32]. They were predicted not to contain the plastid-targeting peptide (predicted by ChloroP v. 1.1 and TargetP prediction programs), suggesting that they function as STSs in *C. angulatus* [33]. As shown in the phylogenetic tree, *CaSTS2*, *CaSTS3,* and *CaSTS4* were assigned to Clade I in the TPS-a subfamily (Figure 8). Clade I is the germacrene A synthase clade, members of which have highly conserved DDxxD and NSE/DTE motifs, responsible for binding of the substrate diphosphate group. Most Clade I STSs catalyze C10-C1 closure to form mono- or double-ring sesquiterpenes. It is widely known that conventional STS functional prediction based on sequence similarities is inaccurate; however, clarifying the functional evolutionary status may provide an alternative strategy for STS functional analysis. According to this, we speculated that these three *CaSTSs* may be 1,10-cyclized STSs, and their mechanism of product formation is similar to the germacrene A synthase.

In order to verify the predicting results, we characterized *CaSTS2*, *CaSTS3,* and *CaSTS4* experimentally, by heterologously expressing these three *CaSTSs* in an engineered *S. cerevisiae* strain LWG003. GC-MS analysis results (Figure S1) showed that *CaSTS2*, *CaSTS3,* and *CaSTS4* catalyzed the formation of germacrene D (C10-C1 closure), similar to our previous study in vitro [33], which is consistent with the functional evolutionary analysis. This demonstrates an application of the structure-based phylogeny in predicting STS function. 

## 3. Discussion

Along with the species evolution, plants have evolved to produce a different collection of terpenenes to accommodate their biotic and abiotic environment [34]. The plant sesquiterpenes gradually formed and might be a potential result of escalating defense and counter-defense between plants and specialized herbivores [35,36]. Exploring the evolutionary origin and history of STSs can not only help to understand the evolutionary pattern and reaction mechanism, but also preliminarily predict the function of STSs. At present, there are many phylogenetic analyses of plant TPSs, most of which focus on taxonomic studies to indicate the evolutionary behavior of TPSs among and within species [16,37], without functional selection of sequences. In this study, all 394 spermatophyte STSs were divided into five distinct groups according to structure-based phylogenetic analysis to explore the evolutionary patterns of plant STSs and sesquiterpenes. 

Many genes are involved in sesquiterpene biosynthesis in the genome of each plant species, which also provides a large platform for the evolution of new sesquiterpenes via gene duplication and subfunctionalization [21,26]. Intron loss, mutations, and coevolution with natural enemies are considered to be the most important evolutionary dynamics of STSs. Evolution in STSs is often the result of intron loss or mutations that lead to subfunctionalization or function loss [38]. For example, a large fragment loss of δ-selinene synthase 2 (*Agsel2*) from *A. grandis* was found around intron X that led to *Agsel2* being transcribed as a pseudogene [39]. Single amino acid W279A switch converts δ-cadinene synthase (CAD1-A) into germacradien-4-ol synthase [40]. New sesquiterpenes keep arising in specific plant lineages, potentially as an outcome of coevolution with natural enemies [35]. Different plant lineages have evolved the ability to make additional “specialized” metabolites that are implicated in defense or the attraction of beneficial organisms, which indicates a dominant process dynamic evolution in STSs to the chemical diversity in plants.

STSs have various evolutionary forms. It can also be expected that STSs with altered subcellular localization and new substrate specificities would have evolved. Although TPSs often have broad substrate specificity and accept GPP, FPP, or GGPP in vitro, their function may be narrower in planta due to their subcellular localization [41]. Monoterpene synthases and diterpene synthases typically contain N-terminus signal peptides and are transported into plastids, STSs, however, are usually found in the cytosol [9]. There is increasing evidence for an exchange of TPS subcellular localization, especially under stress conditions [42,43]. Examples include the *AmNES/LIS-1/2* and *CsLIS/NES-1/2* analyzed above in the TPS-g subfamily. In this scenario, driven by adaptive evolution, ancestral monoterpene synthases losing the N-terminus signal peptide changed their substrate pool and gradually evolved into STSs. Models for gymnosperm TPS evolution proposed that STSs evolved from diterpene synthases through loss of introns, which resulted in, among other changes, the complete loss of the γ domain [39]. Based on this model, *Abies grandis* a-bisabolene synthase *Ag1* (C6-C1 closure), a three-domain plant STSs, is potentially an intermediate in the evolutionary history from diterpene to sesquiterpene synthase [44]. 

Distinct catalytic features of the STSs arose early in spermatophyte evolution and the reactions have become more complex over time. In the evolution of STSs, it is easier to form acyclic sesquiterpenes than cyclic sesquiterpenes according to phylogenetic analysis. Acyclic sesquiterpenes were formed directly from farnesyl cation or nerolidyl cation by proton loss or addition of water [9]. For cyclic sesquiterpenes, they can be formed by typically catalyzing reaction cascades with additional steps, such as the isomerization of carbon–carbon double bond in the initial cation to allow alternate ring closures or additional cyclization [9]. Successive gene duplications and the subsequent accumulation of mutations led to the multitude of STSs, many of which catalyze more complex reactions than the ancestor. Although it is universally accepted that evolution of natural product biosynthesis has led to the formation of more and more complex structures, this process has rarely been documented at the level of a specific enzyme and plant group [45]. Overall, we speculated the early possible evolutionary process of spermatophyte STSs is from acyclic sesquiterpenes to cyclic sesquiterpenes, and the C6-C1 closure sesquiterpenes (small rings) may have formed earlier than C10-C1/C11-C1 closure sesquiterpenes. Interestingly, in some specific STSs, evolution may stop at a certain stage to form a series of characteristic metabolites under certain selective pressures. For example, *Artemisia* species’ STSs are clustered in the A1 clade of the TPS-a subfamily (Figure 7) and obviously originated from a common ancestor dedicated to producing 1,6-cyclized sesquiterpenes. Among these, AaADS from *Artemisia annua* produces the artemisinin specific intermediate amorpha-4,11-diene (C6-C1 cyclized bicyclic sesquiterpenes) [46]; however, other STSs highly homologous to ADS from *Artemisia* species cyclize FPP to (+)-a-bisabolol (C6-C1 monocyclic sesquiterpenes) [47]. 

Systematic study on the evolutionary changes of STS structure is an effective way to elucidate its function. Over the last three decades, high-resolution crystal structures have become available for STSs, and the enzymatic structure–function relationships have revealed the evolutionary relationships of STSs [1,12]. It was recognized early that the structure of STS products depending on the initial substrate conformation imposed by the enzymatic active site cavity [48]. For example, *SaSQS2* is the representative of C6-C1 cyclized STSs, and the shape of FPP conformation is close to natural straight chain in its active site cavity [49]. For C10-C1 cyclized STSs of *TEAS* [50] and *XC1* [51], the shapes of FPP are obviously curved. For the formation of acyclic sesquiterpene, we choose the medium/long-chain-length prenyl pyrophosphate synthase as the representative because there is no crystal structure of acyclic STSs, in which the FPP conformation is almost natural straight chain [52] (Figure S2). Overall, in the evolution of STSs, the change of residues in the active site cavity made the straight chain FPP gradually bend, which made the C1 carbocation gradually approach the intramolecular double bond, and endowed STSs with the ability to form small ring and even large ring sesquiterpenes.

Horizontal gene transfer (HGT) also plays an important role in the evolution of STSs [53]. Sixteen *Santalum* STSs have been functionally charactered (Table S3), among which, 13 STSs are clustered in TPS-b and their products are mainly C6-C1 cyclized sesquiterpene β-bisabolene (Figure 6b), while three STSs are clustered in the R1 clade of the TPS-a subfamily and their products are mainly C10-C1 cyclized sesquiterpenes germacrene D-4-ol, hedycaryol and C11-C1 cyclized sesquiterpene a-humulene (Figure 7). This indicates that the evolutionary sources of STSs in these two parts are completely different. Sandalwood is a semi-parasitic plant, whose survival is inseparable from the host plant. Some studies have shown that sandalwood prefers to parasitize on nitrogen fixing woody plants [54], and HGT events between sandalwood and host plants have also been reported [55,56], which may indicate that STSs in sandalwood come from HGT. These parasitic plants are likely to obtain the synthesis ability of terpenenes and other secondary metabolites from the host through HGT, so as to better adapt to the environment or communicate with the host. 

**Figure 9:**
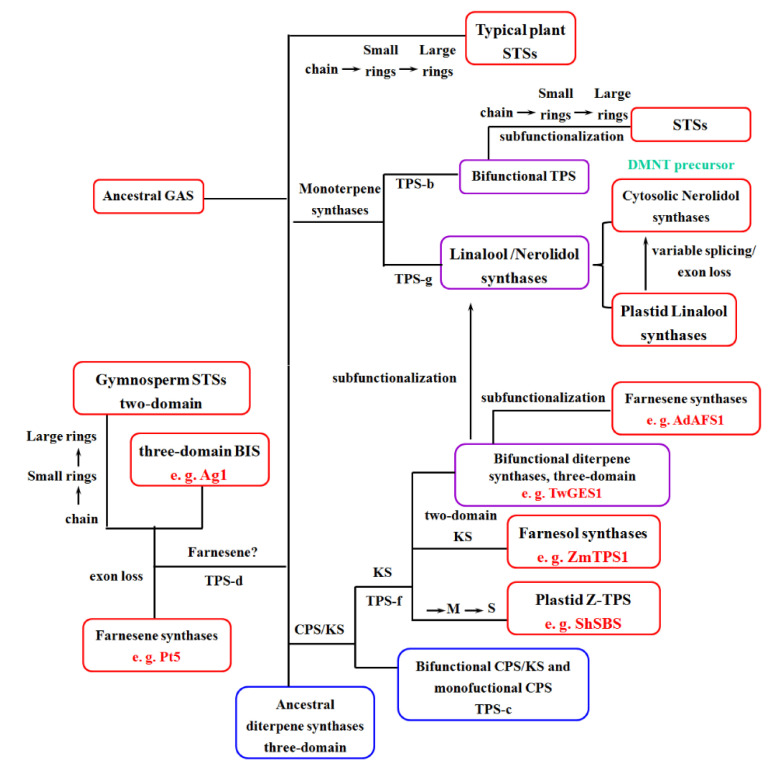
The proposed evolutionary history of spermatophyte STSs. Blue boxes represent ancestral TPSs, red boxes represent STSs, and purple boxes represent transition TPSs. The character “M” represents monoterpene synthases and the character “S” represents STSs. The red words represent the examples of STSs, the green words represent functional products of STSs.

In this comprehensive analysis, on the one hand, we collected the information of plant STSs with identified functions and constructed the phylogeny of plant functional STSs based on the structural comparative analysis, to reveal the sequence–structure–function relationships. On the other hand, we highlighted our incomplete understanding of the evolutionary pattern of sesquiterpenes in spermatophytes (Figure 9), from chain structure to small rings, followed by large rings for the first time, and discussed the evolutionary origins and history of STSs from spermatophyte plants. Then, we proved our evolutionary pattern is useful in predicting the function of STSs by three germacrene D synthases. 

## 4. Materials and Methods

### 4.1. Data Sources and Sequence Retrieval

Data were assembled in two stages: (1) analysis of already annotated STS proteins in public databases and sequence repositories; and (2) mining of novel STS proteins that have no accession numbers from the latest PubMed articles. UniProt database (https://www.uniprot.org/), NCBI (http://www.ncbi.nlm.nih.gov/), and Database of Characterized Plant Sesquiterpene Synthases (http://www.bioinformatics.nl/sesquiterpene/synthasedb/) [15] were the primary source of mining of the STS proteins sequences in 10 January 2021. Only full-length proteins containing all characteristic domains were selected. To find potentially characterized STSs, a detailed retrieval was performed manually for evidence of experimental characterization of sesquiterpenes through in vivo or in vitro GC-MS studies on the PubMed articles, and the corresponding accession numbers were collected. The STS protein information not registered in the public databases was obtained from the autocorrelation literature. 

The sequences collected in this study all showed STS ability in vitro or in the engineered microorganisms, not limited to their actual functions in plants, and all the STS proteins used in this work are listed in Tables S1–S3.

### 4.2. Multiple Sequence Alignment and Phylogeny Construction

Standard approaches were used to reconstruct phylogenetic trees. STSs used for phylogenetic analysis are listed in Tables S1–S3. The selected sequences were aligned by Clustal X 2.0 with default parameters [57], and the alignments were manually fine-tuned afterward. Considering the low similarity of spermatophyte STS sequences, the global phylogenetic analysis was performed by using the maximum likelihood method running 100 bootstrap replications in RaxML v8.2.4 [58]. Phylogenetic analysis of spermatophyte STS with strong genetic relationship was performed by the bootstrap neighbor joining method using MEGA7 [59]. 

### 4.3. Reconstruction of Sequence Similarity Based on Protein Structure

Using the vector alignment search tool (VAST, https://www.ncbi.nlm.nih.gov/Structure/VAST/vastsearch.html) to compare protein structure in 11 March 2021. STSs having complete protein structure were compared with that of TEAS to determine the corresponding relationship on the sequence and the corresponding position of each key site. According to the phylogenetic tree constructed based on the results of sequence alignment, STSs were divided into several groups (based on the evolutionary position and sequence similarity). The sequences in each group were aligned separately, and the key sites and related sequences were manually aligned according to the STSs with identified protein structure in the group. The STSs without identified protein structure were manually adjusted by comparing with TEAS. Finally, the comparison results of each group were combined, and the subsequent evolutionary analysis was carried out. 

### 4.4. Protein Motif and Chloroplast Localization Detection

MEME (Multiple Expectation Maximization for Motif) online tool (https://meme-suite.org/meme/) was used to predict the conserved motifs in spermatophyte STS proteins with default parameters [60] in 11 March 2021, and the protein motifs were visualized with the software TBtools. ChloroP 1.1 online tool (http://www.cbs.dtu.dk/services/ChloroP/) was used to predict the chloroplast localization in 11 March 2021.

### 4.5. Heterologous Expression of CaTPSs in Yeast

An engineered *S. cerevisiae* strain LWG003 [34] (overexpression of *tHMGR, ERG8, ERG10, ERG12, ERG13, ERG19, ERG20, IDI1,* and upc2-1 genes; deletion of *GAL80* gene; and replacement of native promoter of the *ERG9* with the glucose-sensing promoter *HXT1*) was used for in vivo characterization of *CaTPS* genes. The codon optimized genes were synthesized (Genewiz, Nanjing, Suzhou, China) and subcloned into a yeast expression vector pESC-URA. The constructed plasmids were heterologously expressed in LWG003 for the production of sesquiterpenes. An empty vector pESC-URA was also heterologously expressed in Sc027 as control. The plasmids and strains used in this study are listed in Table S4. 

For shake-flask fermentation, colonies of recombinant yeast strains were picked into 10 mL of synthetic medium with 20 g/L glucose, 6.7 g/L yeast nitrogen base, 5 g/L (NH_4_)_2_SO_4_, and 2 g/L amino acid mix lacking uracil and grown at 220 rpm and 30 °C in a shaking incubator for 16-18 h. Subsequently, the culture was inoculated to an initial OD600 of 0.05 from the precultures and then grown for 96 h using the same conditions. Five milliliters of dodecane were added to the 50 mL culture after 10 h for diphasic fermentation. After dehydration with anhydrous magnesium sulfate, the dodecane phase was used for GC-MS (DB-5MS, 30 m × 0.25 mm × 0.25 μm) analysis directly. The injector temperature was 250 °C. One microliter of sample was injected in split mode (1:10), and the GC oven temperature program was applied with 1 mL/min nitrogen as the carrier gas: 70 °C for 2 min, 10 °C/min to 300 °C, and hold for 5 min. The MS scan range (*m*/*z*) was from 35 to 350. The fermentation products were identified based on comparison of their MS spectra and retention times with the NIST17 library.

## Data Availability

Not applicable.

## Supplementary Materials

Supplementary Materials are available online at https://www.mdpi.com/article/10.3390/ijms22126348/s1.

## Abbreviations

STSs	sesquiterpene synthases
FPP	farnesyl diphosphate
TPS	terpenoid synthases
OPP	diphosphate moiety
CPS	copalyl diphosphate synthase
KS	ent-kaurene synthase
DMNT	(*E*)-4,8-dimethyl-1,3,7-nonatriene
TMTT	(*E,E*)-4,8,12-trimethyl-1,3,7,11-tridecatetraene
HGT	horizontal gene transfer
GASs	germacrene A synthases
MEME	Multiple Expectation Maximization for Motif
VAST	vector alignment search tool
