# Supplementary material for: Molecular and Functional Evolution of the Spermatophyte Sesquiterpene Synthases
Source: Int J Mol Sci. 2021 Jun 14;22(12):6348. doi: 10.3390/ijms22126348 (PMC8232007; doi:10.3390/ijms22126348)
Supplement: Supplementary file 1 [file ijms-22-06348-s001.zip › ijms-1225872-SI.pdf]

Supplementary Information

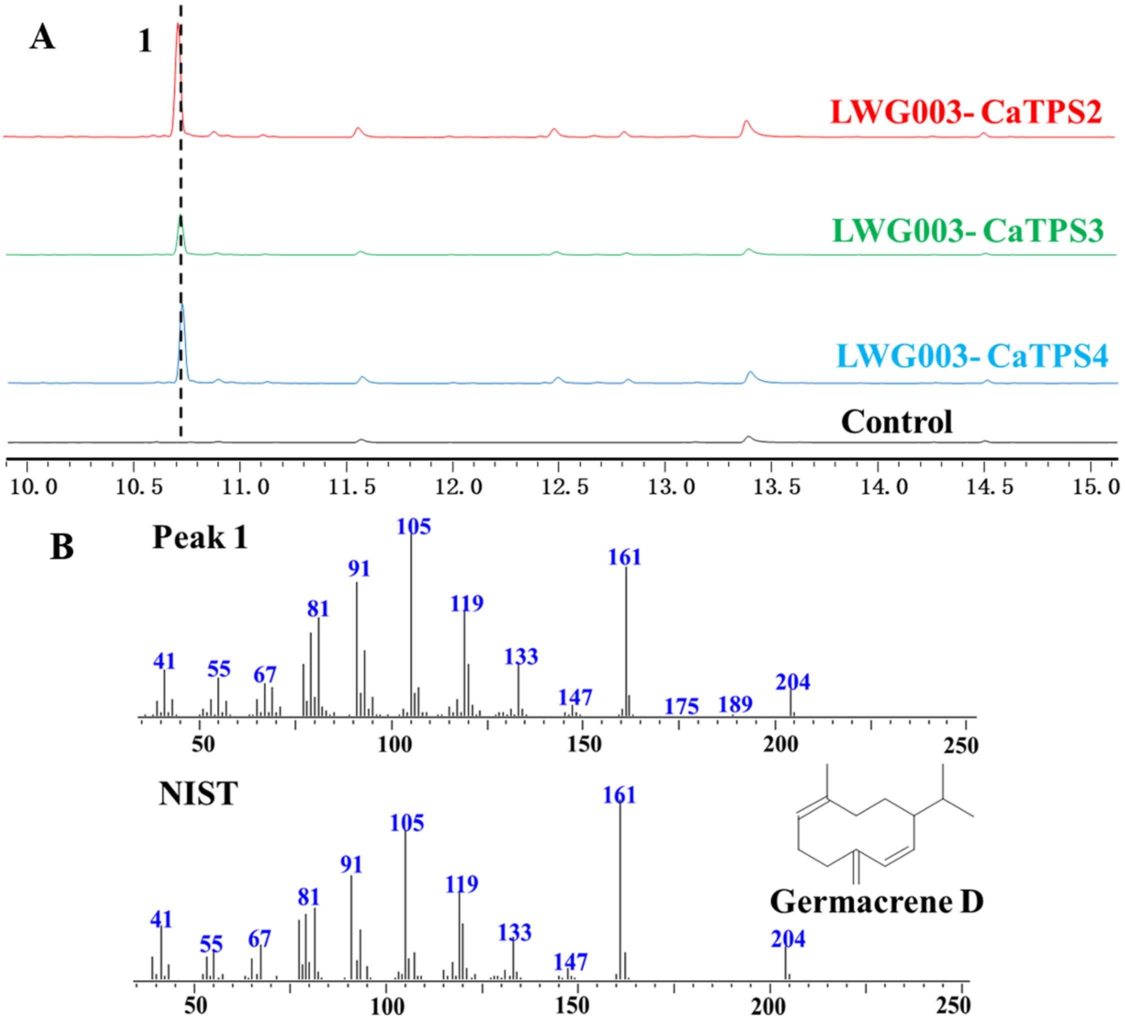

Figure S1. GC–MS analysis of assay products for *CaTPSs* in engineered yeast. (A) GC traces of the fermentation products of LWG003-CaTPS2, LWG003-CaTPS3, LWG003-CaTPS4, and empty vector as control. (B) Mass spectra comparison of the peaks with the compounds stored in the NIST17 library.

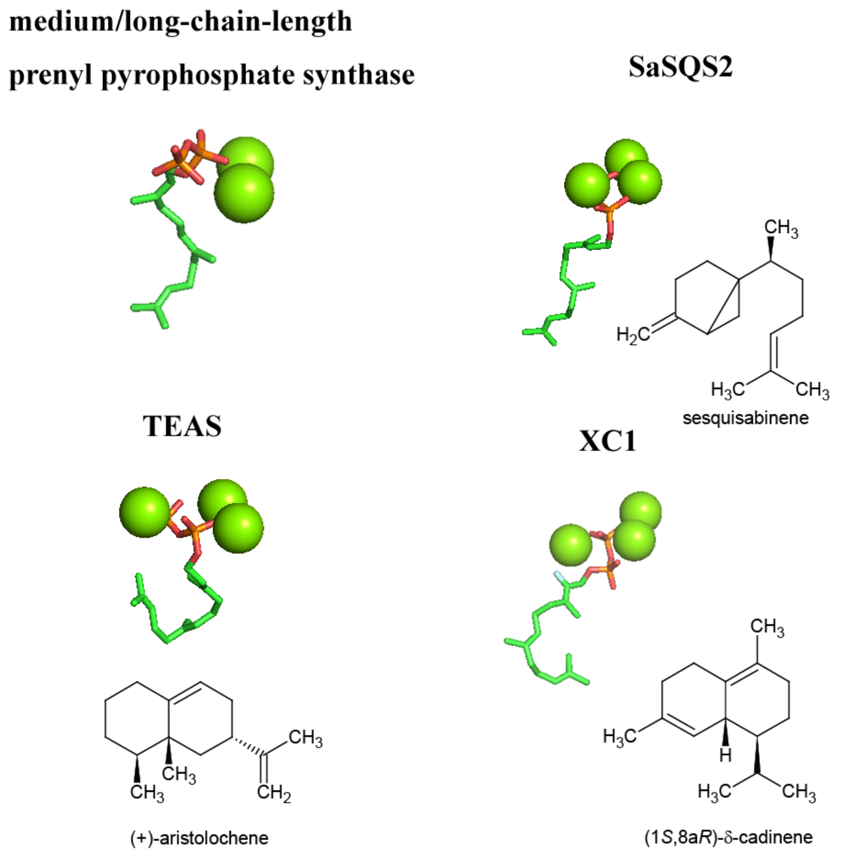

Figure S2. The corresponding shapes of FPP in STSs active site cavity with different cyclization pattern. The medium/long-chain length prenyl pyrophosphate synthase presents the acyclic STSs, SaSQS2 presents the C6-C1 cyclized STSs; TEAS and XC1 present C10-C1 cyclized STSs.

Table S1. Information of functional sesquiterpene TPSs in non-seed plants

Species	Genus	Gene name	GenBank	Products	Function
Myriopteris eatonii	*Polypodiales Pteridaceae Myriopteris*	Mon-GSXD-MTPSL3 [1]	APB88779	(E,E)-α-farnesene	A
Pityrograma trifoliata	*Polypodiales Pteridaceae Pityrogramma*	Mon-UJTT-MTPSL4 [1]	APB88780	protoillud-6-ene	U, T
Woodsia scopulina	*Polypodiales Woodsiaceae Woodsia*	Mon-YJJY-MTPSL1 [1]	APB88781	protoillud-6-ene	U
Selaginella Moellendorffi	*Selaginellales Selaginellaceae Selaginella*	SmMTPSL1 [2]	J9R1J8	-	L
		SmMTPSL17 [2]	D8RLD3	-	M1
		SmMTPSL22 [2]	D8RNZ9	(E)-nerolidol	A, U, B(GPP)
		SmMTPSL26 [2]	J9QS25	2-epi-(E)-β-caryophyllene	C(1,11), M1
Anthoceros punctatus	*Anthocerotales Anthocerotaceae Anthoceros*	Hon-ApMTPSL7 [1]	APB88778	β-acoradiene	C(N1,6), M2, B(GPP)
		ApMTPSL1 [3]	AVL27452	bicyclogermacrene	C(1,10), M1
		ApMTPSL2 [3]	AVL27462	γ-cuprenene	M1, B(GGPP)
		ApMTPSL3 [3]	AVL27453	δ-selinene	C(1,10)
		ApMTPSL4 [3]	AVL27454	-	C(N1,6)
		ApMTPSL6 [3]	AVL27451	β-bisabolene	C(N1,6), B(GGPP)
Anthoceros agrestis		AaMTPSL1 [3]	AVL27456	-	M2
		AaMTPSL3 [3]	AVL27457	δ-selinene	C(1,10)
		AaMTPSL4 [3]	AVL27458	-	C(N1,6), L
		AaMTPSL6 [3]	AVL27460	β-bisabolene	C(N1,6), B(GGPP)
		AaMTPSL7 [3]	AVL27461	β-acoradiene	C(N1,6), M2, B(GPP)
Pseudotaxiphyllum elegans	*Hypnales Hypnaceae Pseudotaxiphyllum*	Mos-QKQO-MTPSL3 [1]	APB88776	γ-curcumene	C(N1,6), M2
Anomodon rostratus	*Hypnales Anomodontaceae Anomodon*	Mos-VBMM-MTPSL3 [1]	APB88777	β-bisabolene	C(N1,6), M1, B(GGPP)
Scapania nemorea	*Jungermanniales Scapaniaceae Scapania*	Liv-IRBN-MTPSL2 [1]	APB88773	bicyclogermacrene	C(1,10), B(GPP)
		Liv-IRBN-MTPSL4 [1]	APB88774	dactylol	M1

Species	Genus	Gene name	GenBank	Products	Function
		MpMTPSL4 [4]	APP91789	5-hydroxy-α-gurjunene	C(N1,10), M1
	Marchantiales	MpMTPSL3 [4]	APP91788	-	B(GPP)
Marchantia polymorpha	*Marchantiaceae*	MpMTPSL5 [4]	APP91790	-	U
	Marchantia	MpMTPSL7 [4]	APP91792	-	M1
		MpMTPSL9 [4]	APP91797	-	M2

Note: C indicates that the main product is cyclized, N indicates that FPP is first isomerized to form NPP and then cyclized, and the number is cyclization mode; A indicates that the main product is acyclized; U indicates single product; M1 indicates multiple products (the number of products is more than 5, and the proportion of main products is less than 50%); M2 indicates that the number of by-products is more than 5, and the proportion of each by-product is less than 1%. B indicates a bifunctional TPS, with other acceptable substrates in brackets; T indicates a tri functional TPS, with GPP, FPP and GGPP as substrates; M indicates monoterpene synthase activity in vivo; D indicates diterpene synthase activity in vivo; L is low TPS activity.

Table S2. Information of functional sesquiterpene TPSs in gymnosperms.

Species	Genus	Gene name	GenBank	Products	Function
Ginkgo biloba	*Ginkgoales Ginkgoaceae Ginkgo*	GbTPS1 [5]	AIU94289	(E,E)-farnesol	A
		GbTPS2 [5]	AIU94290	α-bisabolene	C(N1-6)
Taiwania cryptomerioides	*Cupressales Cupressaceae Taiwania*	TcTPS1 [6]	QGN65607	zingiberene	C(N1-6), M2, B(GPP)
		TcTPS2 [6]	QGN65608	-	C(N1,6), B(GPP)
		TcTPS3 [6]	QGN65609	δ-cadinene	C(N1-10)
		TcTPS4 [6]	QGN65610	germacradien-4-ol	C(N1-10), U
		TcTPS5 [6]	QGN65611	longifolene	C(N1-11), M2
		TcTPS6 [6]	QGN65612	cedrol	C(N1-6), M2, B(GPP)
		TcTPS7 [6]	QGN65613	murrolene or cadinene	C(N1-10), U, B(GPP)
		TcTPS8 [6]	QGN65614	germacrene D	C(N1-10)
		TcTPS9 [6]	QGN65615	caryophyllene	C(1,11)
		TcTPS12 [6]	QHZ00915	δ-cadinene/ germacrene-4-ol	C(N1-10), L
Chamaecyparis formosensis	*Cupressales Cupressaceae Chamaecyparis*	CfCadS [7]	AFJ23663	β-cadinene	C(N1-10)
Pinus sylvestris	*Pinales Pinaceae Pinus*	PsTPS1 [8]	B4XAK4	(E)-β-caryophyllene	C(1,11)
		PsTPS2 [8]	B4XAK5	1(10),5-germacradiene-4-ol	C(1,10), M1
		PsTPS3 [8]	B4XAK6	Longifolene	C(N1-11), M2
Pinus taeda		Pt5 [9]	Q84KL5	α-farnesene	A, U
Abies grandis	*Pinales Pinaceae Abies*	Ag4 [10]	O64404	δ-selinene/(E,E)-Germacrene B	C(1,10), M1, B(GPP)
		Ag5 [10]	O64405	γ-Humulene	C(1,11), M1, B(GPP)
		Ag1 [11]	O81086	(E)-α-bisabolene	C(N1-6), U, B(GPP)
Pseudotsuga menziesii	*Pinales Pinaceae Pseudotsuga*	PmeTPS3 [12]	AAX07266	(Z)-γ-bisabolene	C(N1-6), U
		PmeTPS4 [12]	AAX07265	(E)-β-farnesene	A, U
Picea sitchensis	*Pinales Pinaceae Picea*	PsTPS-Lonp [13]	ADZ45516	α-longipinene	C(N1-11),M1
Picea glauca		PgTPS-Hum [13]	ADZ45513	α-humulene	C(1-11),M1
P. glauca × P. engelmannii		Pg×eTPS-Far/Oci	ADZ45514	(E,E)-α-farnesene	A, U, B(GPP)
		Pg×eTPS-Lonf [13]	ADZ45515	Longifolene	C(N1-11)
Picea abies		PaTPS-Far [14]	AAS47697	E,E-α-Farnesene	A, U
		PaTPS-Lon [14]	AAS47695	Longifolene	C(N1-11), M2
		PaTPS-Bis [14]	AAS47689	E-α-Bisabolene	C(N1-6), U

Note as Table S1.

Table S3. Information of functional sesquiterpene TPSs in angiosperms.

Species	Genus	Gene name	GenBank	Products	Function
	Magnoliales	Mg25 [15]	ACC66281	β-cubebene/α-muurolene	C(N1,10), M1
Magnolia grandiflora	*Magnoliaceae* *Magnolia*	Mg17 [15]	ACC66282	(E)-α-bisabolene	C(N1,6), M1, M
	Magnoliales	CoTPS2 [16]	-	β-cubebene	C(N1,10)
Cananga odorata	*Annonaceae* *Cananga*	CoTPS3 [16]	-	α-bergamotene	C(N1,6)
	Laurales	LnTPS2 [17]	AKQ19358	δ-cadinene/γ-cadinene	C(N1,10), M1
Laurus nobilis	*Lauraceae* *Laurus*	LnTPS3 [17]	AKQ19359	(E,E)-nerolidol	A, D
	Piperales	PnTPS1/PnCPS	ARB08605	caryophyllene	C(1,11)
Piper Nigrum	*Piperales*	PnTPS2/PnCDS [18]	ARB08606	δ-cadinol	C(N1,10)
	Piper	PnTPS3 [18]	AVY53326	Germacrene D	C(N1,10)/(1,10)
	Liliales	LoTPS2 [19]	AMT81306	(E, E)-α-farnesene	A, U
Lilium 'Siberia'	*Liliaceae* *Lilium*	LoTPS4 [19]	-	trans-α-bergamotene	C(N1,6), M
Vanda Mimi Palmer	*Asparagales* *Orchidaceae* *Vanda*	VMPSTS [20]	ABX57720	germacrene D	C(N1,10)/(1,10)
		FhTPS4 [21]	-	Nerolidol	A, U, M
Freesia x hybrida	*Asparagales* *Iridaceae* *Freesia*	FhTPS6 [21]	-	Selinene	C(1,10), B(GPP)
		FhTPS7 [21]	-	Copaene	C(N1,10), M1, B(GPP)
		FhTPS8 [21]	-	α-Gurjunene	C(N1,10), M1
		OsTPS3 [22]	ABJ16553	(E)-β-caryophyllene	C(1,11), M2
		OsTPS13 [22]	ABJ16554	(E,E)-farnesol	A
Oryza sativa		OsLIS [23]	ACF05530	nerolidol	A, U, M
		Os08g07100 [23]	ACF05529	zingiberene	C(N1,6), M1, B(GPP)
	Poales	OsTPS1 [23]	ACF05531	(E)-β-caryophyllene	C(1,11)
Oryza nivara	*Poaceae*	OnTPS1 [24]	AIJ00875	(E)-β-caryophyllene	C(1,11)
Oryza officinalis	*Oryza*	OoTPS1 [24]	AIJ00877	(E)-β-caryophyllene β-elemene	C(1,11)
Oryza glaberrima		OgTPS1[24]	AIJ00879	(E)-β-caryophyllene	C(1,11)
Oryza barthii		ObTPS1[24]	AIJ00878	(E)-β-caryophyllene	C(1,11)
Oryza rufipogon		OrTPS1 [24]	AIJ00876	Germacrene D	C(1,10)
Oryza glumaepatula		OgluTPS1[24]	AIJ00880	(E)-β-caryophyllene	C(1,11)

Species	Genus	Gene name	GenBank	Products	Function
Triticum aestivum	*Poales Poaceae Triticum*	TaPS [25]	-	β -patchoulene	C(1,10)
Eremochloa ophiuroides	*Poales Poaceae Eremochloa*	EoMUS [26]	-	α-muurolene	C(1,10)
Panicum virgatum	*Poales Poaceae Panicum*	PvTPS01 [27]	-	cycloisosativene	-
		PvTPS02 [27]	-	(E)-β-farnesene	A, M2
		PvTPS03 [27]	-	(E)-γ-bisabolene	C(N1,6), M2
		PvTPS05 [27]	-	α- selinene	C(1,10), M1
		PvTPS06 [27]	-	δ- cadinene	C(N1,10), M2
		PvTPS09 [27]	-	δ- cadinene	C(N1,10), M1
		PvTPS10 [27]	-	α-patchoulene	C(1,10), M1
		PvTPS11 [27]	-	(E)-β-caryophyllene	C(1,11)
		PvTPS14 [27]	-	(E)-β-caryophyllene	C(1,11), M1
		PvTPS16 [27]	-	(E)-β-farnesene	A, U
		PvTPS17 [27]	-	β- bisabolene	C(N1,6)
		PvTPS19 [27]	-	(E)-β-caryophyllene	C(1,11)
		PvTPS20 [27]	-	β- bisabolene	C(N1,6), M1
		PvTPS50 [27]	-	copaebe	C(N1,10), M1
		PvTPS55 [27]	-	Germacrene D	C(1,10)/(N1,10)
		PvTPS69 [27]	-	(E)-β-farnesene	A
		PvTPS79 [27]	-	γ-curcumene	C(N1,6), M1
		PvTPS83 [27]	-	(E)-γ-bisabolene	C(N1,6)
		PvTPS94 [27]	-	α-santalene	C(N1,6)
		PvTPS109 [27]	-	(E)-β-farnesene	A, U
Phyllostachys edulis	*Poales Poaceae Phyllostachys*	MoTPS2 [28]	AJP67536	(E, E)-farnesol	A, U
		MoTPS6 [28]	AJP67535	(E)-nerolidol	A, U
Chrysopogon zizanoides	*Poales Poaceae Chrysopogon*	CzZIZS [29]	AJQ30127	(+)-zizaene	C(N1,6)
Ananas comosus	*Poales Bromeliaceae Ananas*	AcoTPS15 [30]	-	clovene	U, B(GPP)

Species	Genus	Gene name	GenBank	Products	Function
Zea mays	*Poales* *Poaceae* *Zea*	ZmTPS23 [31]	B2C4D0	(E)-β-caryophyllene	C(1,11)
		ZmpTPS23 [31]	ABY79213	(E)-β-caryophyllene	C(1,11)
		ZmmTPS23 [31]	ABY79212	(E)-β-caryophyllene	C(1,11)
		ZmhTPS23 [31]	ABY79210	(E)-β-caryophyllene	C(1,11)
		ZmTPS10 [32]	AAX99146	(E)-α–bergamotene,	C(N1,6)
		ZmmTPS10 [32]	ACT37403	(E)-α–bergamotene, (E)-β-farnesene	C(N1,6)
		ZmhTPS10 [32]	ACT37404	(E)-α–bergamotene, (E)-β-farnesene	C(N1,6)
		ZmTPS1 [33]	AAO18435	(E,E)-farnesol	A, B(GPP)
		ZmTPS4-B73 [34]	AAS88571	7-epi-sesquithujene, (S)-β-bisabolene	C(N1,6), M1, B(GPP)
		ZmTPS5-Del1 [34]	AAS88574	Sesquithujene	C(N1,6), M1, B(GPP)
		ZmTPS6 [35]	AAS88576	β-macrocarpene	C(N1,6), B(GPP)
		ZmTPS11 [35]	ACF58240	β-macrocarpene	C(N1,6), B(GPP)
		ZmTPS7 [36]	AAS88577	τ-Cadinol	C(N1,10), M2
		ZmTPS8 [37]	Q29VN3	germacrene D	C(1,10)/(N1,10), M1
		ZmTPS2 [38]	NP_001105854	(E)-nerolidol	A, U, T
		ZmTPS21 [39]	ATI25530	β-selinene	C(1,10)
		ZmEDS [40]	A0A1D6EFT8	eudesmane-2, 11-diol	C(1,10), M1
Zea diploperennis		ZdTPS23 [31]	ABY79209	(E)-β-caryophyllene	C(1,11)
		ZdTPS10 [32]	ACT37405	(E)-β-farnesene	A
Zea perennis		ZpTPS23 [31]	ABY79214	(E)-β-caryophyllene	C(1,11)
		ZpTPS10 [32]	ACT37406	(E)-β-farnesene	A
Zea luxurians		ZlTPS23 [31]	ABY79211	(E)-β-caryophyllene	C(1,11)
Sorghum bicolor	*Poales* *Poaceae* *Sorghum*	SbTPS1 [41]	C5YHH7	Zingiberene	C(N1,6), M2, B(GPP)
		SbTPS2 [41]	C5YHI2	β-sesquiphellandrene	C(N1,6), M2, B(GPP)
		SbTPS1 [41]	C5YHH7	Zingiberene	C(N1,6), M2, B(GPP)
		SbTPS2 [41]	C5YHI2	β-sesquiphellandrene	C(N1,6), M2, B(GPP)
		SbTPS3 [41]	XP_021320232	(E)-β-farnesene	A, M2
		SbTPS4 [41]	-	(E)-β-caryophyllene	C(1,11), M2
		SbTPS5 [41]	-	(Z)-γ-bisabolene	C(N1,6), M1
Zingiber zerumbet	*Zingiberales* *Zingiberaceae* *Zingiber*	ZSS1 [42]	BAG12020	α-humulene	C(1,11)
		ZSS2 [43]	B1B1U4	β-eudesmol	C(1,10)
Zingiber officinale		ZoTPS1 [44]	BAI67934	(S)-β-bisabolene	C(N1,6), U

Species	Genus	Gene name	GenBank	Products	Function
Zingiber officinale	*Zingiberales*<br>*Zingiberaceae*<br>*Zingiber*	ZoGED [45]	AAX40665	(+)-germacrene D	C(1,10), M2
		MT08 [46]	-	(Z)-β-farnesene	A, U, M
		MT06/06A [46]	-	(E)-nerolidol	A, M1, M
		MT02A [46]	-	epi-α-bisabolol	C(N1,6), M
		ST01 [46]	-	β-selinene	C(1,10), M2
		ST05 [46]	-	α-humulene	C(1,11)
		ST05A [46]	-	α-humulene	C(1,11)
		ST07 [46]	-	(-)-caryolan-1-ol	C(1,11)
		ST07A [46]	-	(-)-caryolan-1-ol	C(1,11)
Curcuma longa	*Zingiberales*<br>*Zingiberaceae*<br>*Curcuma*	ClTPS16 [47]	-	β-sesquiphellandrene	C(N1,6)
		ClTPS15 [47]	-	γ-eudesmol	C(1,10), M1
		ClTPS1 [47]	-	-	C(N1,6), M2
		ST00A/B [46]	-	(-)-α-zingiberene	C(N1,6), M2, B(GPP)
		MT00 [46]	-	(E)-nerolidol	A, U, M
		MT17A2 [46]	-	cis-α-bisabolene	C(N1,6), M1, M
		ST02A4 [46]	-	(-)-neointermedeol	C(1,10), M2
		ST02B [46]	-	α-elemol	C(1,10), M2
		ST02C [46]	-	β-elemene	C(1,10), M2
		ST03 [46]	-	γ-amorphene	C(N1,10)
Curcuma zedoaria		CzTPS1a [48]	BBE32333	β-eudesmol	C(1,10)
		CzTps2 [48]	BBE32340	germacrene B	C(1,10), U
Liquidambar formosana	*Saxifragales*<br>*Altingiaceae*<br>*Liquidambar*	LfTPS01 [49]	AIO10964	hedycaryol	C(1,10)
		LfTPS02 [49]	AMD82310	α-selinene	C(1,10)
		LfTPS03 [49]	AIO10966	trans-β-caryophyllene	C(1,11)
		LfTPS04 [49]	AMD82312	δ-cadinene	C(N1,10), M1
		LfTPS05 [49]	AIO10968	nerolidol	A, U, B(GPP)
Medicago Truncatula	*Fabales*<br>*Fabaceae*<br>*Medicago*	MtTPS1 [50]	AAV36464	β-caryophyllene	C(1,11), B(GPP)
		MtTPS3 [50]	AAV36466	(3S) -(E)-nerolidol	A, U, T
		MtTPS5 [50]	ABB01625	(-)-cubebol	C(N1,10), M1, B(GPP)
		MtTPS10 [51]	-	himachalol	C(N1,11), M2
Phaseolus lunatus	*Fabales*<br>*Fabaceae*<br>*Phaseolus*	PlTPS2 [52]	AGS83387	(E)-nerolidol	A, U, T
		PlTPS3 [53]	ARG42161	(E)-nerolidol	A, U, B(GPP)
		PlTPS4 [53]	ARG42162	(E)-nerolidol	A, U, T

Species	Genus	Gene name	GenBank	Products	Function
Sindora glabra	*Fabales* *Fabaceae* *Sindora*	SgSTPS1 [54]	-	β-caryophyllene	C(1,11)
		SgSTPS2 [54]	-	germacrene D	C(N1,10), M1, B(GPP)
Cannabis sativa	*Rosales* *Cannabaceae* *Cannabis*	CsTPS4FN [55]	ARE72260	Alloaromadendrene	C(1,10), B(GPP)
		CsTPS5FN [55]	ARE72256	farnesene	A, U, B(GPP)
		CsTPS7FN [55]	ARE72250	δ-selinene	C(1,10), M1, B(GPP)
		CsTPS8FN [55]	ARE72252	γ-eudesmol, valencene	C(1,10), M1, B(GPP)
		CsTPS9FN [55]	ARE72251	β-caryophyllene	C(1,11)
		CsTPS18VF [56]	QCY41292	(E)-nerolidol	A, U, B(GPP)
		CsTPS19BL [56]	QCY41291	(E)-nerolidol	A, U, B(GPP)
		CsTPS16CC [56]	QCQ18307	GermacreneB	C(1,10), U
		CsTPS20CT [56]	QCY41290	Hedycaryol	C(1,10), U
Humulus lupulus	*Rosales* *Cannabaceae* *Humulus*	HlSTS1 [57]	ACI32639	α-humulene	C(1,11)
		HlSTS2 [57]	ACI32640	germacrene A	C(1,10)
Malus domestica	*Rosales* *Rosaceae* *Malus*	MdAFS [58]	AAO22848	(E,E)-α-farnesene	A
		MdCAR-RG1 [58]	AGB14624	(E)-β-caryophyllene	C(1,11)
		MdGDS-RG1 [58]	AGB14625	(-)-Germacrene D	C(1,10)/(N1,10)
		MdNES-RG1 [58]	AGB14626	(E)-nerolidol	A, U, L
		MdLIS-RG1 [58]	AGB14629	nerolidol	A, U, M
Rosa chinensis	*Rosales* *Rosaceae*	RcLIN-NERS1 [59]	AVR48790	nerolidol	A, B(GPP)
		RcLIN-NERS2 [59]	AVR48791	nerolidol	A, M
Rosa hybrida	*Rosa*	FC0592 [60]	-	Germacrene D	C(1,10)/(N1,10), U
Fragaria ananassa	*Rosales* *Rosaceae* *Fragaria*	FaNES1 [61]	P0CV94	(3S)-E-nerolidol	A, U, B(GPP)
Prunus dulcis	*Rosales* *Rosaceae* *Amygdalus*	PdTPS2 [62]	QEE82240	α-cis-bergamotene	C(N1,6), M1, B(GPP)
		PdTPS4 [62]	QEE82242	α-cis-bergamotene	C(N1,6), M1, B(GPP)
		PdTPS6 [62]	QEE82244	E-α-farnesene	A, B(GPP)
		PdTPS7 [62]	QEE82245	E-nerolidol	A, U, B(GPP)
Cucumis melo	*Cucurbitales* *Cucurbitaceae* *Cucumis*	CmTPSNY [63]	NP_001284382	δ-cadinene	C(N1,10), M2
		CmTPSDul [63]	NP_001284384	α-farnesene	A
Cucumis sativus		CsaFS [64]	AAU05951	(E,E)- α-farnesene	A, U, B(GPP)
		CsbCS [64]	AAU05952	(E)-β-caryophyllene	C(1,11), U

Species	Genus	Gene name	GenBank	Products	Function
Tripterygium wilfordii	*Celastrales Celastraceae Tripterygium*	TwCS [65]	AWV55521	Cryptomeridiol	C(1,10), B(GPP)
		TwTPS12 [66]	APD77393	Elemol	C(1,10)
		TwTPS13 [66]	APD77394	Torreyol	C(N1,10)
		TwTPS20 [66]	ANO43017	Acorenol	C(N1,6), L
		TwTPS22 [66]	ANO43012	Farnesene	A
		TwTPS29 [66]	ANO43019	Nerolidol	A, U
		TwNES [67]	AQA26342	(E)-nerolidol	A, U, B(GGPP)
		TwGES1 [67]	AQA26340	(E)-nerolidol	A, D, L
Populus trichocarpa	*Malpighiales Salicaceae Populus*	PtTPS5 [68]	AII32469	-	C(1,10)
		PtTPS7 [68]	A0A076GAU5	hedycaryol	C(1,10)
		PtTPS9 [68]	AII32473	(E)-β-caryophyllene	C(1,11)
		PtTPS11 [68]	A0A076GAR6	Germacrene A	C(1,10)
		PtTPS12 [68]	AII32476	γ-curcumene	C(N1,6), B(GPP)
		PtTPS15 [68]	AII32468	nerolidol	A, U, B(GPP)
		PtTPS1 [69]	F8TWC9	(-)-germacrene D	C(N1,10), M1
		PtTPS2 [69]	F8TWD0	(E,E)-α–farnesene	A, B(GPP)
		PtTPS3 [69]	F8TWD1	(3S)-nerolidol	A, B(GPP)
		PtTPS4 [69]	F8TWD2	(3S)-nerolidol	A, B(GPP)
		PtdTPS1 [70]	Q64K29	(-)-germacrene D	C(1,10)/(N1,10), M2
Ricinus communis	*Malpighiales Euphorbiaceae Ricinus*	RcSeTPS1 [71]	AEQ27766	(-)-α-copaene	C(N1,10)
		RcSeTPS5 [71]	AEQ27767	-	M1
		RcSeTPS7 [71]	AEQ27768	(E, E)-α-farnesene	A, U
		RcSeTPS10 [71]	AEQ27769	-	C(N1,6), M1
Rhodomyrtus tomentosa	*Myrtales Myrtaceae Rhodomyrtus*	RtTPS1 [72]	AXY92166	β-caryophyllene	C(1,11), B(GPP)
		RtTPS3 [72]	AXY92168	β-caryophyllene	C(1,11), B(GPP)
Pelargonium × hybridum	*Geraniales Geraniaceae Pelargonium*	PhEDS [73]	AWF79083	10-epi-γ-eudesmol	C(1,10)
Murraya koenigii	*Sapindales Rutaceae Murraya*	MkTPS2 [74]	AQT33225	(E,E)-α -farnesene	A, U
Toona sinensis	*Sapindales Meliaceae Toona*	TsTPS2 [75]	BAJ46125	β-elemene	C(1,10), M2

Species	Genus	Gene name	GenBank	Products	Function
Citrus sinensis	*Sapindales*<br>*Rutaceae*<br>*Citrus*	CsTPS1 [76]	AAQ04608	Valencene	C(1,10), U
		CsSQS1 [77]	ATD14141	(Z)-β-cubebene	C(N1,10)
		CsSQS2 [77]	ATD14142	β-cadinene	C(N1,10), M2
		CsSQS3 [77]	ATD14143	β-farnesene	A
		CsSQS4 [77]	ATD14144	Germacrene A	C(1,10), M1
		CsSQS5a/b [77]	ATD14145/<br>ATD14146	alloaromadendrene	C(1,10), M1
		CsSQS6a [77]	ATD14147	β-caryophyllene	C(1,11), L
		CsSQS6b [77]	ATD14148	β-caryophyllene	C(1,11)
		CsSQS7 [77]	ATD14150	β-caryophyllene	C(1,11)
Citrus unshiu		CuSTS4 [78]	BAP75561	nerolidol	A, U, B(GPP)
Citrus jambhiri		RlemTPS4 [79]	BAP74389	δ-elemene	C(1,10), M2, B(GPP)
Citrus junos		CJFS [80]	AAK54279	(E)-β-farnesene	A, U
Zanthoxylum piperitum	*Sapindales*<br>*Rutaceae*<br>*Zanthoxylum*	ZpTPS1 [81]	BBD88588	β-caryophyllene	C(1,11)
		ZpTPS2 [81]	BBD88589	germacrene D	C(1,10)/(N1,10), M2
Arabidopsis Lyrata	*Brassicales*<br>*Brassicaceae*<br>*Arabidopsis*	AlCarS [82]	ACN58564	(E)-β-caryophyllene	C(1,11)
Arabidopsis thaliana		AtTPS24 [83]	Q9LRZ6	(E,E)- α-farnesene	A, U, L, M
		AtTPS14 [83]	Q84UV0	nerolidol	A, U, L, M
		AtTPS21 [83]	Q84UU4	(-)-(E)-β-caryophyllene	C(1,11)
		AtTPS11/At5g44630 [84]	Q4KSH9	(+)-α-barbatene	C(N1,6), M1, B(GPP)
		AtTPS12 [85]	NP_001328007	(Z)-γ-bisabolene	C(N1,6)
		AtTPS13 [85]	AEE83260		
Aquilaria microcarpa	*Malvales*<br>*Thymelaeaceae*<br>*Aquilaria*	dGS-1 [86]	AHH25146	δ-guaiene	C(1,10)
Aquilaria sinensis		ASS1 [87]	AFV99464	δ-guaiene	C(1,10)
		ASS2 [87]	AFV99465	δ-guaiene	C(1,10)
		ASS3 [87]	AFV99466	δ-guaiene	C(1,10)
		As-SesTPS [88]	AGV40227	Nerolidol	A, M2
Aquilaria crassna		AcC2 [89]	ACY38195	δ-guaiene	C(1,10)
		AcC3 [89]	ACY38196	δ-guaiene	C(1,10)
		AcC4 [89]	ACY38197	δ-guaiene	C(1,10)
		AcHS1 [90]	A0A142F308	α-humulene	C(1,11)
		AcHS2 [90]	A0A142F309	α-humulene	C(1,11)
		AcHS3 [90]	A0A142F310	α-humulene	C(1,11)

Species	Genus	Gene name	GenBank	Products	Function
Vitis vinifera	*Vitales* *Vitaceae* *Vitis*	VvValCS [91]	ACO36239	(+)-valencene	C(1,10)
		VvValGW [92]	AAS66358	(+)-valencene	C(1,10)
		VvGerD [92]	AAS66357	(-)-germacrene D	C(N1,10)
		VvGwECar1 [93]	ADR74192	(E)-caryophyllene	C(1,11)
		VvGwECar2 [93]	ADR74193	(E)-caryophyllene	C(1,11)
		VvGwECar3 [93]	ADR74194	(E)-caryophyllene	C(1,11)
		VvPNECar1 [93]	ADR74221	(E)-caryophyllene	C(1,11)
		VvPNECar2 [93]	ADR74222	(E)-caryophyllene	C(1,11)
		VvGwGerA [93]	ADR66821	germacrene A	C(1,10)
		VvGwaBer [93]	ADR74195	(E)-α-bergamotene	C(N1,6)
		VvGwGerD [93]	ADR74196	germacrene D	C(1,10)
		VvPNGerD [93]	ADR74197	germacrene D	C(1,10)
		VvCSaFar [93]	ADR74198	(E,E)-α-farnesene	A, U
		VvGwgCad [93]	ADR74199	γ-cadinene	C(N1,10)
		VvPNbCur [93]	ADR74200	(E)-γ-bisabolene	C(N1,6), M1
		VvPNSesq [93]	ADR74223	Sesquithujene	C(N1,6), M2
		VvPNaZin [93]	ADR74224	α-zingiberene	C(N1,6)
		VvPNSeInt [93]	CAO39293	selina-4,11-diene,	C(1,10), M1
		VvPNCuCad [93]	ADR74226	Cubebol, δ-cadinene	C(N1,10), M1
		VvPNaHum [93]	ADR74227	α-humulene	C(1,11)
		VvPNEb2epi Car [93]	ADR74228	(E)-β-caryophyllene	C(1,11)
		VvGwbOciF/ VvCSbOciF [93]	ADR74207/ ADR74208	(E,E)-α-farnesene	A, U, M
		VvPNLinNer1 [93]	ADR74210	(E)-Nerolidol	A, U, B(GPP)
		VvPNLinNer2 [93]	ADR74211	(E)-Nerolidol	A, U, B(GPP)
		VvCSLinNer [93]	ADR74212	(E)-Nerolidol	A, U, B(GPP)
		VvPNLNGl1 [93]	ADR74213	(E)-Nerolidol	A, U, T
		VvPNLNGl2 [93]	ADR74214	(E)-Nerolidol	A, U, T
		VvPNLNGl3 [93]	ADR74215	(E)-Nerolidol	A, U, T
		VvPNLNGl4 [93]	ADR74216	(E)-Nerolidol	A, U, T
		VvCSENerGl [93]	ADR74219	(E)-Nerolidol	A, U, B(GGPP)
		VvPNENerGl [93]	ADR74220	(E)-Nerolidol	A, U, B(GGPP)
		VvShTPS07 [94]	NP_001268063	Ylangene	C(N1,10), M1
		VvShTPS26 [94]	XP_002283070	α-Cubebene	C(N1,10), M1

Species	Genus	Gene name	GenBank	Products	Function
		VvShTPS27 [94]	XP_019072407	Isocaryophyllene	C(1,11)
Cistus Creticus Subsp. Creticus	*Malvales Cistaceae Cistus*	CcGer [95]	ACF94469	germacrene B	C(1,10)
Gossypium hirsutum	*Malvales Malvaceae Gossypium*	GhTPS1 [96]	AGX84975	β-caryophyllene	C(1,11)
		GhTPS2 [96]	AGX84976	guaia-1(10),11-diene	C(1,10)
Gossypium arboreum		XC1/XC14 [97]	AAA93064	(+)-δ-Cadinene	C(N1,10), U
		CAD1-A [98]	NP_001316949	(+)-δ-Cadinene	C(N1,10), U
Santalum album	*Santalales Santalaceae Santalum*	SaSQS1 [99]	AIV42939	Sesquisabinene	C(N1,6), B(GPP)
		SaSQS2 [99]	AIV42940	Sesquisabinene	C(N1,6), B(GPP)
		SaBS [99]	AIV42941	(S)-β-bisabolene	C(N1,6), B(GPP)
		SaSS [99]	AGV01243	α-santalene	C(N1,6), M1
		SaSSy [99]	E3W202	α-santalene	C(N1,6), M1
		SamonoTPS1 [100]	ACF24767	β-bisabolene	C(N1,6), U, M
		SaSesquiTPS1 [100]	ACF24768	germacrene D-4-ol	C(1,10), M1, B(GPP)
		SaTPS2[101]	AZM65215	sesquisabinene	C(N1,6), M1, B(GPP)
		SaTPS3 [101]	AZM65216	(E)-nerolidol	A, M1, B(GPP)
Santalum spicatum		SspiTPS4 [102]	AJG35803	sesquisabinene B	C(N1,6), B(GPP)
		SspiSSy [103]	ADO87002	α-santalene	C(N1,6)
		SspiBS [103]	ADO87004	β-bisabolene	C(N1,6)
		SspiSesquiTPS [103]	ADO87006	hedycaryol	C(1,10)
Santalum austrocaledonicum		SauSSy [103]	ADO87001	α-santalene	C(N1,6)
		SauBS [103]	ADO87003	β-bisabolene	C(N1,6), B(GPP)
		SauSesquiTPS [103]	ADO87005	a-humulene	C(1,11), M2
Persicaria minor	*Caryophyllales Polygonaceae Persicaria*	PmSTS [104]	AFN57631	β-sesquiphellandrene	C(N1,6)
		PmSTPS1 [105]	-	α -farnesene	A, M1
		PmSTPS2 [105]	AWK77755	nerolidol	A
Persicaria hydropiper		PhDS [106]	AHF22834	drimenol	U
Camellia sinensis	*Ericales Theaceae Camellia*	CsNES [107]	ARQ20729	(E)-nerolidol	A, U, B(GPP)
		CsLIS/NES-1 [108]	AGX26045	(E)-nerolidol	A, U, M
		CsLIS/NES-2 [108]	-	(E)-nerolidol	A, U, B(GPP)
		CsAFS [109]	-	α -farnesene	A, U, B(GPP)
Camellia brevistyla		CbTPS1 [110]	A0A140KFG9	hedycaryol	C(1,10), U
Actinidia deliciosa	*Ericales Actinidiaceae*	AdAFS1 [111]	ACO40485	(E,E)-α-farnesene	A, B(GPP)
		AdGDS1[111]	AAX16121	(+)-germacrene D	C(1,10)/(N1,10), U

Species	Genus	Gene name	GenBank	Products	Function
Actinidia chinensis	*Actinidia*	AcNES1 [112]	AER36088	(S)-(E)-nerolidol	A, T
Ocimum basilicum	*Lamiales Lamiaceae Ocimum*	ObSES [113]	AAV63785	β-selinene	C(1,10), M1
		ObCDS [113]	Q5SBP5	γ-cadinene	C(N1,10)
		ObZIS [113]	AAV63788	α-zingiberene	C(N1,6), M2, B(GPP)
		ObGDS [113]	AAV63786	germacrene D	C(1,10)/(N1,10)
Ocimum kilimandscharicum		OkBCS [113]	AKA94109	β-caryophyllene	C(1,11)
Lavandula angustifolia	*Lamiales Lamiaceae Lavandula*	LaBERS [114]	ABB73046	trans-α-bergamotene	C(N1,6), B(GPP)
		LaGERDS [115]	AGL98420	germacrene D	C(1,10)/(N1,10)
		LaCARS [115]	AGL98419	(E)-β-caryophyllene	C(1,11), U, B(GPP)
		LaCADS [115]	AGL98418	τ-cadinol	C(N1,10)
Lavandula X Intermedia		LiCPS [116]	AGU13712	9-epi-caryophyllene	C(1,11), U, B(GPP)
Lavandula pedunculata		LpGEAS [117]	AGN72800	germacrene A	C(1,10), U
Lavandula stoechas		LsGEAS [117]	AGN72803	germacrene A	C(1,10), U
Lavandula viridis		LvGEAS [117]	AGN72806	germacrene A	C(1,10), U
Leucosceptrum canum	*Lamiales Lamiaceae Leucosceptrum*	Lc-CedS [118]	QBP05430	cedrol	C(N1,6)
Origanum vulgare	*Lamiales Lamiaceae Origanum*	OvTPS3 [119]	ADK73619	(-)-germacrene D	C(1,10)/(N1,10), B(GPP)
		OvTPS4 [119]	ADK73618	alloaromadendrene	C(1,10), B(GPP)
		OvTPS6-d06 [119]	ADK73616	(E)-β-caryophyllene	C(1,11)
		OvTPS6-f02 [119]	ADK73615	(E)-β-caryophyllene	C(1,11)
Mentha x piperita	*Lamiales Lamiaceae Mentha*	MxpFAS [120]	O48935	(E)-β-farnesene	A, B(GPP)
		MxpSS1 [121]	Q5W283	cis-muurola-3,5-diene, cis-muurola-4(14),5-diene	C(N1,10)
Pogostemon cablin	*Lamiales Lamiaceae Pogostemon*	PatTpsA [122]	AAS86319	γ-curcumene	C(N1,6), L
		PatTpsBF2 [122]	AAS86320	(-)-germacrene D	C(1,10), L
		PatTpsCF2 [122]	AAS86321	(+)-germacrene A	C(1,10)
		PatTpsB15 [122]	AAS86322	(-)-germacrene D	C(1,10), M2
		PatTps177 [122]	Q49SP3	(-)-patchoulol	C(1,10), M2
		PTSiso [123]	AAS86323	germacrene A	C(1,10), M1
Osmanthus fragrans	*Lamiales Oleaceae Osmanthus*	OfTPS4 [124]	A0A109QXM3	α-farnesene	A, U

Species	Genus	Gene name	GenBank	Products	Function
Antirrhinum majus	*Lamiales Plantaginaceae Antirrhinum*	AmNES/LIS-1 [125]	ABR24417	(3S)(E)-nerolidol	A, B(GPP)
		AmNES/LIS-2 [125]	ABR24418	(3S)(E)-nerolidol	A, U, M
Phyla dulcis/ Lippia Dulcis	*Lamiales Verbenaceae Phyla /Lippia*	LdTPS1 [126]	J7LP58	α-Copaene、δ-cadinene	C(N1,10)
		LdTPS5 [126]	J7LMP2	bicyclogermacrene	C(1,10)
		LdTPS6 [126]	J7LJN5	β-caryophyllene	C(1,11), U
		LdTPS7 [126]	J7LQ09	trans-α-bergamotene	C(N1,6)
		LdTPS8 [126]	J7LH11	α-bisabolol	C(N1,6), U
Solanum habrochaites	*Solanales Solanaceae Solanum*	ShSBS [127]	ACJ38409	(+)-endo-β-bergamotene	C(N1,6), B(GPP)
		ShTPS9 [128]	AEM23825	germacrene B	C(1,10), B(GPP)
		ShTPS12 [128]	AEM23826	α-humulene	C(1,11), B(GPP)
		ShTPS17 [128]	AEM23828	valencene	C(1,10), B(GPP)
		ShTPS15b [128]	AEM23830	germacrene A	C(1,10)
		ShTPS14a [128]	AEM23827	(Z)-α-bisabolene	C(N1,6), B(GPP), M1
		ShTPS14b [128]	AEM23829	β-bisabolene	C(N1,6), M1
		SSTLH1 [129]	Q9FQ27	germacrene B	C(1,10), U
		SSTLH2 [129]	AAG41892	Germacrene D	C(1,10)
		ShZIS [130]	AFJ67794	7-epizingiberene	C(N1,6)
Solanum lycopersicum		SlTPS9(STLE1) [128]	AEM05858	germacrene C	C(1,10), B(GPP)
		SlTPS12 [128]	AEP82783	β-caryophyllene	C(1,11), B(GPP)
		SlTPS17 [128]	AEP82780	valencene	C(1,10), B(GPP)
		SlTPS14 [128]	AEP82782	(Z)-γ-bisabolene	C(N1,6), B(GPP)
		SlTPS31 [128]	AEM23833	viridiflorene	C(1,10), U
		SlTPS36 [131]	AEP82770	-	-
		SlTPS32 [131]	AEP82773	viridiflorene	C(1,10)
		SlTPS38 [131]	AEP82768	α-bergamotene	C(N1,6)
		SlTPS37 [131]	AEP82769	nerolidol	A, B(GPP)
		SlTPS39 [131]	AEP82767	nerolidol	A, B(GPP)
		SSTLE2 [129]	AAG41890	δ-elemene	C(1,10)
		SlTPS5(LeMTS1) [131]	AAX69063	(E)-nerolidol	A, U, M
Lycopersicon esculentum cv. VFNT		pLE11.3 [132]	-	germacrene C	C(1,10), B(GPP)
		pLE14.2 [132]	AAC39432	germacrene C	C(1,10)
		pLE20.3 [132]	AAC39431	germacrene C	C(1,10)
Solanum elaeagnifolium		Cl7653 [133]	-	β-caryophyllene	C(1,11)

Species	Genus	Gene name	GenBank	Products	Function
Hyoscyamus muticus	*Solanales* *Solanaceae* *Hyoscyamus*	HuVS1 [134]	Q39978	vetispiradiene	C(1,10), U
		HuVS2 [134]	Q39979	vetispiradiene	C(1,10), U
		HuVS3 [134]	Q39980	vetispiradiene	C(1,10), U
Capsicum annuum	*Solanales* *Solanaceae* *Capsicum*	PEAS [135]	AAC61260	5-epi-aristolochene	C(1,10), U
Nicotiana tabacum	*Solanales* *Solanaceae* *Nicotiana*	EAS110 [136]	AAP79448	5-epi-aristolochene	C(1,10), U
		TEAS [137]	Q40577	5-epi-aristolochene	C(1,10)
Nicotiana attenuata		NaEAS12 [138]	Q84LF2	5-epi-aristolochene	C(1,10), U
		NaEAS34 [138]	Q84LF1	5-epi-aristolochene	C(1,10), U
		NaEAS37 [138]	Q84LF0	5-epi-aristolochene	C(1,10), U
Matricaria recutita	*Asterales* *Asteraceae* *Matricaria*	MrTPS1 [139]	A0A075W0Z3	(−)-α-bisabolol	C(N1,6), U
		MrTPS4 [139]	A0A075W3D9	Bicyclogermacrene	C(1,10), U
		MrTPS6 [139]	A0A075W2D6	β-farnesene	A, U
		MrTPS1′ [140]	AFM43734	(-)-(E)-β-caryophyllene	C(1,11)
		MrTPS2′ [140]	AFM43735	α-isocomene	C(1,11)
		MrTPS3′ [140]	I6QSN0	(+)-germacrene A	C(1,10), U
		MrTPS5′ [140]	AFM43738	(-)-germacrene D	C(1,10)/(N1,10), M2
		Mr-bFS [141]	AIW60869	(E)-β-farnesene	A, U
Solidago Canadensis	*Asterales* *Asteraceae* *Solidago*	Sc1 [142]	CAC36896	(+)-(10R)-germacrene A	C(1,10)
		Sc19 [143]	Q70EZ6	(-)-germacrene D	C(1,10)
		Sc11 [143]	Q70EZ7	(+)-germacrene D	C(1,10)
Ixeris dentate	*Asterales* *Asteraceae* *Ixeris*	IdGAS [144]	AAL92481	(+)-germacrene A	C(1,10), U
Barnadesia spinosa	*Asterales* *Asteraceae* *Barnadesia*	BsGAS1 [145]	AIX97167	germacrene A	C(1,10), U
		BsGAS2 [145]	AIX97168	germacrene A	C(1,10), U
Taraxacum officinale	*Asterales* *Asteraceae* *Taraxacum*	ToGAS1 [146]	ALY05868	(+)-germacrene A	C(1,10), U
		ToGAS2 [146]	ALY05869	(+)-germacrene A	C(1,10), U
Tanacetum cinerariifolium	*Asterales* *Asteraceae* *Tanacetum*	TcGAS [147]	AGO03788	germacrene A	C(1,10), U
Tanacetum parthenium		TpGAS [148]	AEH41844	germacrene A	C(1,10), U

Species	Genus	Gene name	GenBank	Products	Function
		TpCarS [148]	AEH41845	b-caryophyllene	C(1,11)
Eremanthus erythropappus	*Asterales<br>Asteraceae<br>Eremanthus*	EeBOS1 [149]	AYJ71561	(-)-α-bisabolol	C(N1,6), U
Cichorium intybus	*Asterales<br>Asteraceae<br>Cichorium*	CiGASsh [150]	Q8LSC2	(+)-germacrene A	C(1,10), U
		CiGASlo [150]	Q8LSC3	(+)-germacrene A	C(1,10), U
Lactuca sativa	*Asterales<br>Asteraceae<br>Lactuca*	LTC1 [151]	AAM11626	germacrene A	C(1,10), U
		LTC2 [151]	AAM11627	germacrene A	C(1,10), U
Helianthus annuus	*Asterales<br>Asteraceae<br>Helianthus*	HaTPS12-K7 [152]	AME16497	(Z)-γ-bisabolene	C(N1,6), U
		HaTPS12-K11 [152]	AME16498	(Z)-γ-bisabolene	C(N1,6), U
		HaGAS1 [153]	AAY41421	germacrene A	C(1,10), U
		HaGAS2 [153]	ABY49939	germacrene A	C(1,10), U
		HaTPS2 (HaCS) [153]	ACA33926	δ-cadinene	C(N1,10), M1
		HaGAS3 [154]	ACZ50512	germacrene A	C(1,10)
Xanthium Strumarium	*Asterales<br>Asteraceae<br>Xanthium*	XsTPS1[155]	AMP42987	germacrene D	C(1,10)/(N1,10)
		XsTPS2 [155]	AMP42991	guaia-4,6-diene	C(1,10), U
		XsTPS3 [155]	AJT60315	germacrene A	C(1,10), U
Centaurea stoebe	*Asterales<br>Asteraceae<br>Centaurea*	CsTPS1 [156]	-	α-muurolene	C(N1,10), U
		CsTPS4 [156]	-	(E)-β-caryophyllene	C(1,11)
		CsTPS5 [156]	-	daucadiene	M2
		CsTPS7 [156]	-	(E)-α-bisabolene	C(N1,6)
		CsTPS8 [156]	-	α-zingiberene	C(N1,6), L
Achillea millefolium	*Asterales<br>Asteraceae<br>Achillea*	AmGAS [157]	AGD80135	Germacrene A	C(1,10),B(GPP)
Senecio scandens	*Asterales<br>Asteraceae<br>Senecio*	SsNES [158]	MH518312	(E)-nerolidol	A, U
Cynara cardunculus	*Asterales<br>Asteraceae<br>Cynara*	CcGAS [159]	AET95645	(+) germacrene A	C(1,10), U
Daucus carota	*Apiales<br>Apiaceae*	DcTPS1 [160]	XP_017214942	(E)-β-caryophyllene	C(1,11), B(GPP)

Daucus

Species	Genus	Gene name	GenBank	Products	Function
Artemisia absinthium		AabKOS [161]	A0A1L7NYG2	Koidzumiol	C(N1,6), U
Artemisia kurramensis		AkBOS [161]	A0A1L7NYG3	(+)-α-Bisabolol	C(N1,6), U
Artemisia maritima		AmBOS [161]	A0A1L7NYF8	(+)-α-Bisabolol	C(N1,6), U
		AmAOS [162]	BBG75037	4-amorphen-11-ol	C(N1,6)
Artemisia annua		AaBOS [163]	M4GGS0	α-bisabolol	C(N1,6), M2
		AaGAS [164]	ABE03980	germacrene A	C(1,10), U
	Asterales<br>*Asteraceae*<br>*Artemisia*	QHS1 [165]	AAL79181	β-caryophyllene	C(1,11)
		AaADS [166]	AFA34434	amorpha-4,11-diene	C(N1,6), M2
		AaECS1 [167]	CAC08805	epi-cedrol	C(N1,6), M2, B(GPP)
		AaBFS [168]	AAX39387	(E)-β-farnesene	A, U
Artemisia abrotanum		AabrBOS1 [169]	-	(+)-α-bisabolol	C(N1,6), U
		AabrBOS2 [169]	-	(+)-α-bisabolol	C(N1,6), U
		AabrBOS3 [169]	-	(+)-α-bisabolol	C(N1,6), U
		AabrBOS4 [169]	-	(+)-α-bisabolol	C(N1,6), U
		AabrSPS [169]	-	7-epi-silphiperfol-5-ene	C(1,11), U
Thapsia garganica	*Apiales*<br>*Apiaceae*<br>*Thapsia*	TgTPS1 [170]	AFV09098	trans-nerolidol	A
		TgTPS2 [170]	AFV09099	kunzeaol	C(1,10), M2
Thapsia laciniata		TlTPS820 [171]	AZL40026	epikunzeaol	C(1,10), U
		TlTPS509 [171]	AZL40028	Guaiol	C(1,10), M1
		TlTPS18983 [171]	AZL40027	farnesene	A, M1
		TlTPS7414 [172]	AMU19318	(−)-germacrene D	C(1,10)/(N1,10)
Acanthopanax sciadophylloides	*Apiales*<br>*Araliaceae*<br>*Acanthopanax*	AscTps1 [48]	BBE32330	β-caryophyllene	C(1,11)
		AscTps2a [48]	BBE32331	germacrene D	C(1,10)/(N1,10), U
		AscTPS3 [48]	BBE32339	nerolidol	A, B(GPP)
Acanthopanax sieboldianus		AsiTPS1a [48]	BBE32335	β-caryophyllene	C(1,11)
Valeriana Officinalis	*Dipsacales*<br>*Caprifoliaceae*<br>*Valeriana*	VoTPS1′ [173]	AFR42417	germacrene C	C(1,10)
		VoTPS2′ [173]	AFR42418	valerena-4,7(11)-diene	C(1,10)
		VoTPS1 [174]	AGB05610	valerena-1,10-diene	C(1,10), B(GPP)
		VoTPS7 [174]	AGB05616	germacrene C	C(1,10), B(GPP)
		VoTPS3′ [175]	AFR42419	(-)-drimenol	U

Note as Table S1.

Table S4. Plasmids and strains used in this study

Name	Description	Resource
	Plasmids	
pESC-URA	2 μ, URA3	stored in the lab
pESCU-CaTPS2	cloning *CaTPS2* into pESC-URA	this study
pESCU-CaTPS3	cloning *CaTPS3* into pESC-URA	this study
pESCU-CaTPS4	cloning *CaTPS4* into pESC-URA	stored in the lab
	Strains	
LWG003	CEN.PK2-1C derivative; leu2-3,112:: G418^R_T_{ERG19}(RC)-ERG19(RC)-P_{GAL1}(RC)_P_{GAL10}-ERG8-T_{ERG8}; his3Δ1:: HIS3 _T_{ERG12}(RC)-ERG12(RC)-P_{GAL1}(RC)_P_{GAL10}-ERG10-T_{ERG10}; ade1Δ:: T_{HMG1}(RC)-tHMG1(RC)-P_{GAL1}(RC)_P_{GAL10}-IDI1-T_{IDI1}_ADE1; ura3-52:: T_{HMG1}(RC)-tHMG1(RC)-P_{GAL1}(RC)_P_{GAL10}-ERG13-T_{ERG13}; trp1-289:: T_{HMG1}(RC)-tHMG1(RC)-P_{GAL1}(RC)_P_{GAL10}-ERG20-T_{ERG20}_TRP1; ΔGAL80::P_{GAL2}-upc2-1-T_{ADH1}; ΔPERG9:: PHXT1	stored in the lab
LWG003- CaTPS2	LWG003, pESCU-CaTPS2	this study
LWG003- CaTPS3	LWG003, pESCU-CaTPS3	this study
LWG003- CaTPS4	LWG003, pESCU-CaTPS4	this study

References

1. Jia, Q.; Li, G.; Kollner, T.G.; Fu, J.; Chen, X.; Xiong, W.; Crandall-Stotler, B.J.; Bowman, J.L.; Weston, D.J.; Zhang, Y.; et al. Microbial-type terpene synthase genes occur widely in nonseed land plants, but not in seed plants. *P Natl Acad Sci USA* **2016**, *113*, 12328-12333, doi:10.1073/pnas.1607973113.
2. Li, G.; Koellner, T.G.; Yin, Y.; Jiang, Y.; Chen, H.; Xu, Y.; Gershenzon, J.; Pichersky, E.; Chen, F. Nonseed plant Selaginella moellendorfii has both seed plant and microbial types of terpene synthases. *P Natl Acad Sci USA* **2012**, *109*, 14711-14715, doi:10.1073/pnas.1204300109.
3. Xiong, W.; Fu, J.; Koellner, T.G.; Chen, X.; Jia, Q.; Guo, H.; Qian, P.; Guo, H.; Wu, G.; Chen, F. Biochemical characterization of microbial type terpene synthases in two closely related species of hornworts, Anthoceros punctatus and Anthoceros agrestis. *Phytochemistry* **2018**, *149*, 116-122, doi:10.1016/j.phytochem.2018.02.011.
4. Kumar, S.; Kempinski, C.; Zhuang, X.; Norris, A.; Mafu, S.; Zi, J.; Bell, S.A.; Nybo, S.E.; Kinison, S.E.; Jiang, Z.; et al. Molecular Diversity of Terpene Synthases in the Liverwort Marchantia polymorpha. *Plant Cell* **2016**, *28*, 2632-2650, doi:10.1105/tpc.16.00062.
5. Parveen, I.; Wang, M.; Zhao, J.; Chittiboyina, A.G.; Tabanca, N.; Ali, A.; Baerson, S.R.; Techen, N.; Chappell, J.; Khan, I.A.; et al. Investigating sesquiterpene biosynthesis in Ginkgo biloba: molecular cloning and functional characterization of (E,E)-farnesol and alpha-bisabolene synthases. *Plant Molecular Biology* **2015**, *89*, 451-462, doi:10.1007/s11103-015-0381-3.
6. Ma, L.-T.; Lee, Y.-R.; Liu, P.-L.; Cheng, Y.-T.; Shiu, T.-F.; Tsao, N.-W.; Wang, S.-Y.; Chu, F.-H. Phylogenetically distant group of terpene synthases participates in cadinene and cedrane-type sesquiterpenes accumulation in Taiwania cryptomerioides. *Plant Science* **2019**, *289*, doi:10.1016/j.plantsci.2019.110277.
7. Kuo, P.-M.; Hsu, K.-H.; Lee, Y.-R.; Chu, F.-H.; Wang, S.-Y. Isolation and characterization of beta-cadinene synthase cDNA from Chamaecyparis formosensis Matsum. *Holzforschung* **2012**, *66*, 569-576, doi:10.1515/hf-2011-0224.
8. Koepke, D.; Schroeder, R.; Fischer, H.M.; Gershenzon, J.; Hilker, M.; Schmidt, A. Does egg deposition by herbivorous pine sawflies affect transcription of sesquiterpene synthases in pine? *Planta* **2008**, *228*, 427-438, doi:10.1007/s00425-008-0747-8.
9. Phillips, M.A.; Wildung, M.R.; Williams, D.C.; Hyatt, D.C.; Croteau, R. cDNA isolation, functional expression, and characterization of (+)-alpha-pinene synthase and (-)-alpha-pinene synthase from loblolly pine (Pinus taeda): Stereocontrol in pinene biosynthesis. *Archives of Biochemistry and Biophysics* **2003**, *411*, 267-276, doi:10.1016/s0003-9861(02)00746-4.
10. Steele, C.L.; Crock, J.; Bohlmann, J.; Croteau, R. Sesquiterpene synthases from grand fir (Abies grandis) - Comparison of constitutive and wound-induced activities, and cdna isolation, characterization and bacterial expression of delta-selinene synthase and gamma-humulene synthase. *Journal of Biological Chemistry* **1998**, *273*, 2078-2089, doi:10.1074/jbc.273.4.2078.
11. Bohlmann, J.; Crock, J.; Jetter, R.; Croteau, R. Terpenoid-based defenses in conifers: cDNA cloning, characterization, and functional expression of wound-inducible (E)-alpha-bisabolene synthase from grand fir (Abies grandis). *P Natl Acad Sci USA* **1998**, *95*, 6756-6761, doi:10.1073/pnas.95.12.6756.
12. Huber, D.P.W.; Philippe, R.N.; Godard, K.A.; Sturrock, R.N.; Bohlmann, J. Characterization of four terpene synthase cDNAs from methyl jasmonate-induced Douglas-fir, Pseudotsuga menziesii. *Phytochemistry* **2005**, *66*, 1427-1439, doi:10.1016/j.phytochem.2005.04.030.
13. Keeling, C.I.; Weisshaar, S.; Ralph, S.G.; Jancsik, S.; Hamberger, B.; Dullat, H.K.; Bohlmann, J. Transcriptome mining, functional characterization, and phylogeny of a large terpene synthase gene family in spruce (Picea spp.). *Bmc Plant Biology* **2011**, *11*, doi:10.1186/1471-2229-11-43.
14. Martin, D.M.; Faldt, J.; Bohlmann, J. Functional characterization of nine Norway spruce TPS genes and evolution of gymnosperm terpene synthases of the TPS-d subfamily. *Plant Physiology* **2004**, *135*, 1908-1927, doi:10.1104/pp.104.042028.
15. Lee, S.; Chappell, J. Biochemical and genomic characterization of terpene synthases in Magnolia grandiflora. *Plant Physiology* **2008**, *147*, 1017-1033, doi:10.1104/pp.108.115824.
16. Jin, J.; Kim, M.J.; Dhandapani, S.; Tjhang, J.G.; Yin, J.-L.; Wong, L.; Sarojam, R.; Chua, N.-H.; Jang, I.-C. The floral transcriptome of ylang ylang (Cananga odorata var. fruticosa) uncovers biosynthetic pathways for volatile organic compounds and a multifunctional and novel sesquiterpene synthase. *Journal of Experimental Botany* **2015**, *66*, 3959-3975, doi:10.1093/jxb/erv196.
17. Yahyaa, M.; Matsuba, Y.; Brandt, W.; Doron-Faigenboim, A.; Bar, E.; McClain, A.; Davidovich-Rikanati, R.; Lewinsohn, E.; Pichersky, E.; Ibdah, M. Identification, Functional Characterization, and Evolution of

Terpene Synthases from a Basal Dicot. *Plant Physiology* **2015**, *169*, 1683-1697, doi:10.1104/pp.15.00930.
18. Jin, Z.; Kwon, M.; Lee, A.-R.; Ro, D.-K.; Wungsintaweekul, J.; Kim, S.-U. Molecular cloning and functional characterization of three terpene synthases from unripe fruit of black pepper (Piper nigrum). *Archives of Biochemistry and Biophysics* **2018**, *638*, 35-40, doi:10.1016/j.abb.2017.12.011.
19. Abbas, F.; Ke, Y.; Zhou, Y.; Ashraf, U.; Li, X.; Yu, Y.; Yue, Y.; Ahmad, K.W.; Yu, R.; Fan, Y. Molecular cloning, characterization and expression analysis of LoTPS2 and LoTPS4 involved in floral scent formation in oriental hybrid Lilium variety 'Siberia'. *Phytochemistry* **2020**, *173*, doi:10.1016/j.phytochem.2020.112294.
20. Song, A.A.L.; Abdullah, J.O.; Abdullah, M.P.; Shafee, N.; Rahim, R.A. Functional Expression of an Orchid Fragrance Gene in Lactococcus lactis. *International Journal of Molecular Sciences* **2012**, *13*, 1582-1597, doi:10.3390/ijms13021582.
21. Gao, F.; Liu, B.; Li, M.; Gao, X.; Fang, Q.; Liu, C.; Ding, H.; Wang, L.; Gao, X. Identification and characterization of terpene synthase genes accounting for volatile terpene emissions in flowers of Freesia x hybrida. *Journal of Experimental Botany* **2018**, *69*, 4249-4265, doi:10.1093/jxb/ery224.
22. Cheng, A.-X.; Xiang, C.-Y.; Li, J.-X.; Yang, C.-Q.; Hu, W.-L.; Wang, L.-J.; Lou, Y.-G.; Chen, X.-Y. The rice (E)-beta-caryophyllene synthase (OsTPS3) accounts for the major inducible volatile sesquiterpenes. *Phytochemistry* **2007**, *68*, 1632-1641, doi:10.1016/j.phytochem.2007.04.008.
23. Yuan, J.S.; Koellner, T.G.; Wiggins, G.; Grant, J.; Degenhardt, J.; Chen, F. Molecular and genomic basis of volatile-mediated indirect defense against insects in rice. *Plant Journal* **2008**, *55*, 491-503, doi:10.1111/j.1365-313X.2008.03524.x.
24. Chen, H.; Li, G.; Koellner, T.G.; Jia, Q.; Gershenzon, J.; Chen, F. Positive Darwinian selection is a driving force for the diversification of terpenoid biosynthesis in the genus Oryza. *Bmc Plant Biology* **2014**, *14*, doi:10.1186/s12870-014-0239-x.
25. Pu, Q.; Liang, J.; Shen, Q.; Fu, J.; Pu, Z.; Liu, J.; Wang, X.; Wang, Q. A Wheat beta-Patchoulene Synthase Confers Resistance against Herbivory in Transgenic Arabidopsis. *Genes* **2019**, *10*, doi:10.3390/genes10060441.
26. Lee, G.W.; Chung, M.-S.; Lee, S.S.; Chung, B.Y.; Lee, S. Transcriptome-guided identification and functional characterization of key terpene synthases involved in constitutive and methyl jasmonate-inducible volatile terpene formation in Eremochloa ophiuroides (Munro) Hack. *Plant Physiology and Biochemistry* **2019**, *141*, 193-201, doi:10.1016/j.plaphy.2019.05.032.
27. Muchlinski, A.; Chen, X.; Lovell, J.T.; Koellner, T.G.; Pelot, K.A.; Zerbe, P.; Ruggiero, M.; Callaway, L., III; Laliberte, S.; Chen, F.; et al. Biosynthesis and Emission of Stress-Induced Volatile Terpenes in Roots and Leaves of Switchgrass (Panicum virgatum L.). *Frontiers in Plant Science* **2019**, *10*, doi:10.3389/fpls.2019.01144.
28. Chen, X.; Wang, Y.; Sun, J.; Wang, J.; Xun, H.; Tang, F. Cloning, expression and functional characterization of two sesquiterpene synthase genes from moso bamboo (Phyllostachys edulis). *Protein Expression and Purification* **2016**, *120*, 1-6, doi:10.1016/j.pep.2015.11.019.
29. Hartwig, S.; Frister, T.; Alemdar, S.; Li, Z.; Scheper, T.; Beuterl, S. SUMO-fusion, purification, and characterization of a (+)-zizaene synthase from Chrysopogon zizanioides. *Biochemical and Biophysical Research Communications* **2015**, *458*, 883-889, doi:10.1016/j.bbrc.2015.02.053.
30. Chen, X.; Yang, W.; Zhang, L.; Wu, X.; Cheng, T.; Li, G. Genome-wide identification, functional and evolutionary analysis of terpene synthases in pineapple. *Computational Biology and Chemistry* **2017**, *70*, 40-48, doi:10.1016/j.compbiolchem.2017.05.010.
31. Kollner, T.G.; Held, M.; Lenk, C.; Hiltpold, I.; Turlings, T.; Gershenzon, J.; Degenhardt, J. A maize (E)-beta-caryophyllene synthase implicated in indirect defense responses against herbivores is not expressed in most American maize varieties. *The Plant Cell* **2008**, *20*, 482-494.
32. Koellner, T.G.; Gershenzon, J.; Degenhardt, J. Molecular and biochemical evolution of maize terpene synthase 10, an enzyme of indirect defense. *Phytochemistry* **2009**, *70*, 1139-1145, doi:10.1016/j.phytochem.2009.06.011.
33. Schnee, C.; Kollner, T.G.; Gershenzon, J.; Degenhardt, J. The maize gene terpene synthase 1 encodes a sesquiterpene synthase catalyzing the formation of (E)-beta-farnesene, (E)-nerolidol, and (E,E)-farnesol after herbivore damage. *Plant Physiology* **2002**, *130*, 2049-2060, doi:10.1104/pp.008326.
34. Kollner, T.G.; Schnee, C.; Gershenzon, J.; Degenhardt, J. The variability of sesquiterpenes cultivars is controlled by allelic emitted from two Zea mays variation of two terpene synthase genes encoding stereoselective multiple product enzymes. *Plant Cell* **2004**, *16*, 1115-1131, doi:10.1105/tpc.019877.
35. Koellner, T.G.; Schnee, C.; Li, S.; Svatos, A.; Schneider, B.; Gershenzon, J.; Degenhardt, J. Protonation of a neutral (S)-beta-bisabolene intermediate is involved in (S)-beta-macrocarpene formation by the maize sesquiterpene synthases TPS6 and TPS11. *Journal of Biological Chemistry* **2008**, *283*, 20779-20788,

doi:10.1074/jbc.M802682200.
36. Ren, F.; Mao, H.; Liang, J.; Liu, J.; Shu, K.; Wang, Q. Functional characterization of ZmTPS7 reveals a maize tau-cadinol synthase involved in stress response. *Planta* **2016**, *244*, 1065-1074, doi:10.1007/s00425-016-2570-y.
37. Fontana, A.; Held, M.; Fantaye, C.A.; Turlings, T.C.; Degenhardt, J.; Gershenzon, J. Attractiveness of Constitutive and Herbivore-Induced Sesquiterpene Blends of Maize to the Parasitic Wasp Cotesia marginiventris (Cresson). *Journal of Chemical Ecology* **2011**, *37*, 582-591, doi:10.1007/s10886-011-9967-7.
38. Richter, A.; Schaff, C.; Zhang, Z.; Lipka, A.E.; Tian, F.; Kollner, T.G.; Schnee, C.; Preiss, S.; Irmisch, S.; Jander, G.; et al. Characterization of Biosynthetic Pathways for the Production of the Volatile Homoterpenes DMNT and TMTT in Zea mays. *Plant Cell* **2016**, *28*, 2651-2665, doi:10.1105/tpc.15.00919.
39. Ding, Y.; Huffaker, A.; Kollner, T.G.; Weckwerth, P.; Robert, C.A.M.; Spencer, J.L.; Lipka, A.E.; Schmelz, E.A. Selinene Volatiles Are Essential Precursors for Maize Defense Promoting Fungal Pathogen Resistance. *Plant Physiology* **2017**, *175*, 1455-1468, doi:10.1104/pp.17.00879.
40. Liang, J.; Liu, J.; Brown, R.; Jia, M.; Zhou, K.; Peters, R.J.; Wang, Q. Direct production of dihydroxylated sesquiterpenoids by a maize terpene synthase. *Plant Journal* **2018**, *94*, 847-856, doi:10.1111/tpj.13901.
41. Zhuang, X.; Koellner, T.G.; Zhao, N.; Li, G.; Jiang, Y.; Zhu, L.; Ma, J.; Degenhardt, J.; Chen, F. Dynamic evolution of herbivore-induced sesquiterpene biosynthesis in sorghum and related grass crops. *Plant Journal* **2012**, *69*, 70-80, doi:10.1111/j.1365-313X.2011.04771.x.
42. Yu, F.; Okamto, S.; Nakasone, K.; Adachi, K.; Matsuda, S.; Harada, H.; Misawa, N.; Utsumi, R. Molecular cloning and functional characterization of alpha-humulene synthase, a possible key enzyme of zerumbone biosynthesis in shampoo ginger (Zingiber zerumbet Smith). *Planta* **2008**, *227*, 1291-1299, doi:10.1007/s00425-008-0700-x.
43. Yu, F.; Harada, H.; Yamasaki, K.; Okamoto, S.; Hirase, S.; Tanaka, Y.; Misawa, N.; Utsumi, R. Isolation and functional characterization of a beta-eudesmol synthase, a new sesquiterpene synthase from Zingiber zerumbet Smith. *Febs Letters* **2008**, *582*, 565-572, doi:10.1016/j.febslet.2008.01.020.
44. Fujisawa, M.; Harada, H.; Kenmoku, H.; Mizutani, S.; Misawa, N. Cloning and characterization of a novel gene that encodes (S)-beta-bisabolene synthase from ginger, Zingiber officinale. *Planta* **2010**, *232*, 121-130, doi:10.1007/s00425-010-1137-6.
45. Picaud, S.; Olsson, M.E.; Brodelius, M.; Brodelius, P.E. Cloning, expression, purification and characterization of recombinant (+)-germacrene D synthase from Zingiber officinale. *Archives of Biochemistry and Biophysics* **2006**, *452*, 17-28, doi:10.1016/j.abb.2006.06.007.
46. Koo, H.J.; Gang, D.R. Suites of Terpene Synthases Explain Differential Terpenoid Production in Ginger and Turmeric Tissues. *Plos One* **2012**, *7*, doi:10.1371/journal.pone.0051481.
47. Sun, J.; Cui, G.; Ma, X.; Zhan, Z.; Ma, Y.; Teng, Z.; Gao, W.; Wang, Y.; Chen, T.; Lai, C.; et al. An integrated strategy to identify genes responsible for sesquiterpene biosynthesis in turmeric. *Plant Molecular Biology* **2019**, *101*, 221-234, doi:10.1007/s11103-019-00892-0.
48. Hattan, J.-i.; Shindo, K.; Sasaki, T.; Misawa, N. Isolation and Functional Characterization of New Terpene Synthase Genes from Traditional Edible Plants. *Journal of Oleo Science* **2018**, *67*, 1235-1246, doi:10.5650/jos.ess18163.
49. Chuang, L.; Wen, C.-H.; Lee, Y.-R.; Lin, Y.-L.; Hsu, L.-R.; Wang, S.-Y.; Chu, F.-H. Identification, Functional Characterization, and Seasonal Expression Patterns of Five Sesquiterpene Synthases in Liquidambar formosana. *Journal of Natural Products* **2018**, *81*, 1162-1172, doi:10.1021/acs.jnatprod.7b00773.
50. Arimura, G.-I.; Garms, S.; Maffei, M.; Bossi, S.; Schulze, B.; Leitner, M.; Mithofer, A.; Boland, W. Herbivore-induced terpenoid emission in Medicago truncatula: concerted action of jasmonate, ethylene and calcium signaling. *Planta* **2008**, *227*, 453-464, doi:10.1007/s00425-007-0631-y.
51. Yadav, H.; Dreher, D.; Athmer, B.; Porzel, A.; Gavrin, A.; Baldermann, S.; Tissier, A.; Hause, B. Medicago TERPENE SYNTHASE 10 Is Involved in Defense Against an Oomycete Root Pathogen. *Plant Physiology* **2019**, *180*, 1598-1613, doi:10.1104/pp.19.00278.
52. Brillada, C.; Nishihara, M.; Shimoda, T.; Garms, S.; Boland, W.; Maffei, M.E.; Arimura, G.-i. Metabolic engineering of the C-16 homoterpene TMTT in Lotus japonicus through overexpression of (E,E)-geranyllinalool synthase attracts generalist and specialist predators in different manners. *New Phytologist* **2013**, *200*, 1200-1211, doi:10.1111/nph.12442.
53. Li, F.; Li, W.; Lin, Y.-J.; Pickett, J.A.; Birkett, M.A.; Wu, K.; Wang, G.; Zhou, J.-J. Expression of lima bean terpene synthases in rice enhances recruitment of a beneficial enemy of a major rice pest. *Plant Cell and Environment* **2018**, *41*, 111-120, doi:10.1111/pce.12959.
54. Yu, N.; Yang, J.-C.; Yin, G.-T.; Li, R.-S.; Zou, W.-T. Transcriptome Analysis of Oleoresin-Producing Tree Sindora Glabra and Characterization of Sesquiterpene Synthases. *Frontiers in Plant Science* **2018**, *9*,

doi:10.3389/fpls.2018.01619.
55. Booth, J.K.; Page, J.E.; Bohlmann, J. Terpene synthases from Cannabis sativa. *Plos One* **2017**, *12*.
56. Zager, J.J.; Lange, I.; Srividya, N.; Smith, A.; Lange, B.M. Gene Networks Underlying Cannabinoid and Terpenoid Accumulation in Cannabis. *Plant Physiology* **2019**, *180*, 1877-1897, doi:10.1104/pp.18.01506.
57. Wang, G.; Tian, L.; Aziz, N.; Broun, P.; Dai, X.; He, J.; King, A.; Zhao, P.X.; Dixon, R.A. Terpene Biosynthesis in Glandular Trichomes of Hop. *Plant Physiology* **2008**, *148*, 1254-1266, doi:10.1104/pp.108.125187.
58. Nieuwenhuizen, N.J.; Green, S.A.; Chen, X.; Bailleul, E.J.D.; Matich, A.J.; Wang, M.Y.; Atkinson, R.G. Functional Genomics Reveals That a Compact Terpene Synthase Gene Family Can Account for Terpene Volatile Production in Apple. *Plant Physiology* **2013**, *161*, 787-804, doi:10.1104/pp.112.208249.
59. Magnard, J.-L.; Bony, A.R.; Bettini, F.; Campanaro, A.; Blerot, B.; Baudino, S.; Jullien, F. Linalool and linalool nerolidol synthases in roses, several genes for little scent. *Plant Physiology and Biochemistry* **2018**, *127*, 74-87, doi:10.1016/j.plaphy.2018.03.009.
60. Guterman, I.; Shalit, M.; Menda, N.; Piestun, D.; Dafny-Yelin, M.; Shalev, G.; Bar, E.; Davydov, O.; Ovadis, M.; Emanuel, M.; et al. Rose scent: Genomics approach to discovering novel floral fragrance-related genes. *Plant Cell* **2002**, *14*, 2325-2338, doi:10.1105/tpc.005207.
61. Aharoni, A.; Giri, A.P.; Verstappen, F.W.A.; Bertea, C.M.; Sevenier, R.; Sun, Z.K.; Jongsma, M.A.; Schwab, W.; Bouwmeester, H.J. Gain and loss of fruit flavor compounds produced by wild and cultivated strawberry species. *Plant Cell* **2004**, *16*, 3110-3131, doi:10.1105/tpc.104.023895.
62. Nawade, B.; Yahyaa, M.; Reuveny, H.; Shaltiel-Harpaz, L.; Eisenbach, O.; Faigenboim, A.; Bar-Yaakov, I.; Holland, D.; Ibdah, M. Profiling of volatile terpenes from almond (Prunus dulcis) young fruits and characterization of seven terpene synthase genes. *Plant Science* **2019**, *287*, doi:10.1016/j.plantsci.2019.110187.
63. Portnoy, V.; Benyamini, Y.; Bar, E.; Harel-Beja, R.; Gepstein, S.; Giovannoni, J.J.; Schaffer, A.A.; Burger, J.; Tadmor, Y.; Lewinsohn, E.; et al. The molecular and biochemical basis for varietal variation in sesquiterpene content in melon (Cucumis melo L.) rinds. *Plant Molecular Biology* **2008**, *66*, 647-661, doi:10.1007/s11103-008-9296-6.
64. Mercke, P.; Kappers, I.F.; Verstappen, F.W.A.; Vorst, O.; Dicke, M.; Bouwmeester, H.J. Combined transcript and metabolite analysis reveals genes involved in spider mite induced volatile formation in cucumber plants. *Plant Physiology* **2004**, *135*, 2012-2024, doi:10.1104/pp.104.048116.
65. Tong, Y.-r.; Su, P.; Guan, H.-y.; Hu, T.-y.; Chen, J.-l.; Zhang, Y.-f.; Zhao, Y.-j.; Gao, L.-h.; Zhang, X.-n.; Huang, L.-q.; et al. Eudesmane-type sesquiterpene diols directly synthesized by a sesquiterpene cyclase in Tripterygium wilfordii. *Biochemical Journal* **2018**, *475*, 2713-2725, doi:10.1042/bcj20180353.
66. Hansen, N.L.; Heskes, A.M.; Hamberger, B.; Olsen, C.E.; Hallstrom, B.M.; Andersen-Ranberg, J.; Hamberger, B. The terpene synthase gene family in Tripterygium wilfordii harbors a labdane-type diterpene synthase among the monoterpene synthase TPS-b subfamily. *Plant Journal* **2017**, *89*, 429-441, doi:10.1111/tpj.13410.
67. Su, P.; Hu, T.; Liu, Y.; Tong, Y.; Guan, H.; Zhang, Y.; Zhou, J.; Huang, L.; Gao, W. Functional characterization of NES and GES responsible for the biosynthesis of (E)-nerolidol and (E, E)-geranyllinalool in Tripterygium wilfordii. *Scientific Reports* **2017**, *7*, doi:10.1038/srep40851.
68. Irmisch, S.; Jiang, Y.; Chen, F.; Gershenzon, J.; Koellner, T.G. Terpene synthases and their contribution to herbivore-induced volatile emission in western balsam poplar (Populus trichocarpa). *Bmc Plant Biology* **2014**, *14*, doi:10.1186/s12870-014-0270-y.
69. Danner, H.; Boeckler, G.A.; Irmisch, S.; Yuan, J.S.; Chen, F.; Gershenzon, J.; Unsicker, S.B.; Koellner, T.G. Four terpene synthases produce major compounds of the gypsy moth feeding-induced volatile blend of Populus trichocarpa. *Phytochemistry* **2011**, *72*, 897-908, doi:10.1016/j.phytochem.2011.03.014.
70. Arimura, G.; Huber, D.P.W.; Bohlmann, J. Forest tent caterpillars (Malacosoma disstria) induce local and systemic diurnal emissions of terpenoid volatiles in hybrid poplar (Populus trichocarpa x deltoides): cDNA cloning, functional characterization, and patterns of gene expression of (-)-germacrene D synthase, PtdTPS1. *Plant Journal* **2004**, *37*, 603-616, doi:10.1111/j.1365-313X.2003.01987.x.
71. Xie, X.; Kirby, J.; Keasling, J.D. Functional characterization of four sesquiterpene synthases from Ricinus communis (Castor bean). *Phytochemistry* **2012**, *78*, 20-28, doi:10.1016/j.phytochem.2012.02.022.
72. He, S.-M.; Wang, X.; Yang, S.-C.; Dong, Y.; Zhao, Q.-M.; Yang, J.-L.; Cong, K.; Zhang, J.-J.; Zhang, G.-H.; Wang, Y.; et al. De novo Transcriptome Characterization of Rhodomyrtus tomentosa Leaves and Identification of Genes Involved in alpha/beta-Pinene and beta-Caryophyllene Biosynthesis. *Frontiers in Plant Science* **2018**, *9*, doi:10.3389/fpls.2018.01231.
73. Blerot, B.; Martinelli, L.; Prunier, C.; Saint-Marcoux, D.; Legrand, S.; Bony, A.; Sarrabere, L.; Gros, F.; Boyer, N.; Caissard, J.-C.; et al. Functional Analysis of Four Terpene Synthases in Rose-Scented Pelargonium

Cultivars (Pelargonium x hybridum) and Evolution of Scent in the Pelargonium Genus. *Frontiers in Plant Science* **2018**, *9*, doi:10.3389/fpls.2018.01435.
74. Meena, S.; Kumar, S.R.; Dwivedi, V.; Singh, A.K.; Chanotiya, C.S.; Akhtar, M.Q.; Kumar, K.; Shasany, A.K.; Nagegowda, D.A. Transcriptomic insight into terpenoid and carbazole alkaloid biosynthesis, and functional characterization of two terpene synthases in curry tree (Murraya koenigii). *Scientific Reports* **2017**, *7*, doi:10.1038/srep44126.
75. Hsu, C.-Y.; Huang, P.-L.; Chen, C.-M.; Mao, C.-T.; Chaw, S.-M. Tangy Scent in Toona sinensis (Meliaceae) Leaflets: Isolation, Functional Characterization, and Regulation of TsTPS1 and TsTPS2, Two Key Terpene Synthase Genes in the Biosynthesis of the Scent Compound. *Current Pharmaceutical Biotechnology* **2012**, *13*, 2721-2732, doi:10.2174/138920112804724864.
76. Sharon-Asa, L.; Shalit, M.; Frydman, A.; Bar, E.; Holland, D.; Or, E.; Lavi, U.; Lewinsohn, E.; Eyal, Y. Citrus fruit flavor and aroma biosynthesis: isolation, functional characterization, and developmental regulation of Cstps1, a key gene in the production of the sesquiterpene aroma compound valencene. *Plant Journal* **2003**, *36*, 664-674, doi:10.1046/j.1365-313X.2003.01910.x.
77. Alquezar, B.; Rodriguez, A.; de la Pena, M.; Pena, L. Genomic Analysis of Terpene Synthase Family and Functional Characterization of Seven Sesquiterpene Synthases from Citrus sinensis. *Frontiers in Plant Science* **2017**, *8*, doi:10.3389/fpls.2017.01481.
78. Shimada, T.; Endo, T.; Fujii, H.; Rodriguez, A.; Pena, L.; Omura, M. Characterization of three linalool synthase genes from Citrus unshiu Marc. and analysis of linalool-mediated resistance against Xanthomonas citri subsp citri and Penicilium italicum in citrus leaves and fruits. *Plant Science* **2014**, *229*, 154-166, doi:10.1016/j.plantsci.2014.09.008.
79. Uji, Y.; Ozawa, R.; Shishido, H.; Taniguchi, S.; Takabayashi, J.; Akimitsu, K.; Gomi, K. Isolation of a sesquiterpene synthase expressing in specialized epithelial cells surrounding the secretory cavities in rough lemon (Citrus jambhiri). *Journal of Plant Physiology* **2015**, *180*, 67-71, doi:10.1016/j.jplph.2015.03.013.
80. Maruyama, T.; Ito, M.; Honda, G. Molecular cloning, functional expression and characterization of (E)-beta-farnesene synthase from Citrus junos. *Biological & Pharmaceutical Bulletin* **2001**, *24*, 1171-1175, doi:10.1248/bpb.24.1171.
81. Fujita, Y.; Koeduka, T.; Aida, M.; Suzuki, H.; Iijima, Y.; Matsui, K. Biosynthesis of volatile terpenes that accumulate in the secretory cavities of young leaves of Japanese pepper (Zanthoxylum piperitum): Isolation and functional characterization of monoterpene and sesquiterpene synthase genes. *Plant Biotechnology* **2017**, *34*, 17-+, doi:10.5511/plantbiotechnology.16.1231a.
82. Abel, C.; Clauss, M.; Schaub, A.; Gershenzon, J.; Tholl, D. Floral and insect-induced volatile formation in Arabidopsis lyrata ssp petraea, a perennial, outcrossing relative of A.thaliana. *Planta* **2009**, *230*, 1-11, doi:10.1007/s00425-009-0921-7.
83. Chen, F.; Tholl, D.; D'Auria, J.C.; Farooq, A.; Pichersky, E.; Gershenzon, J. Biosynthesis and emission of terpenoid volatiles from Arabidopsis flowers. *Plant Cell* **2003**, *15*, 481-494, doi:10.1105/tpc.007989.
84. Tholl, D.; Chen, F.; Petri, J.; Gershenzon, J.; Pichersky, E. Two sesquiterpene synthases are responsible for the complex mixture of sesquiterpenes emitted from Arabidopsis flowers. *Plant Journal* **2005**, *42*, 757-771, doi:10.1111/j.1365-313X.2005.02417.x.
85. Ro, D.-K.; Ehlting, J.; Keeling, C.I.; Lin, R.; Mattheus, N.; Bohlmann, J. Microarray expression profiling and functional characterization of AtTPS genes: Duplicated Arabidopsis thaliana sesquiterpene synthase genes At4g13280 and At4g13300 encode root-specific and wound-inducible (Z)-gamma-bisabolene synthases. *Archives of Biochemistry and Biophysics* **2006**, *448*, 104-116, doi:10.1016/j.abb.2005.09.019.
86. Lee, J.-B.; Hirohashi, S.; Yamamura, Y.; Taura, F.; Kurosaki, F. Induction, Cloning and Functional Expression of a Sesquiterpene Biosynthetic Enzyme, delta-Guaiene Synthase, of Aquilaria microcarpa Cell Cultures. *Natural Product Communications* **2014**, *9*, 1231-1235.
87. Xu, Y.; Zhang, Z.; Wang, M.; Wei, J.; Chen, H.; Gao, Z.; Sui, C.; Luo, H.; Zhang, X.; Yang, Y.; et al. Identification of genes related to agarwood formation: transcriptome analysis of healthy and wounded tissues of Aquilaria sinensis. *Bmc Genomics* **2013**, *14*, doi:10.1186/1471-2164-14-227.
88. Ye, W.; He, X.; Wu, H.; Wang, L.; Zhang, W.; Fan, Y.; Li, H.; Liu, T.; Gao, X. Identification and characterization of a novel sesquiterpene synthase from Aquilaria sinensis: An important gene for agarwood formation. *International Journal of Biological Macromolecules* **2018**, *108*, 884-892, doi:10.1016/j.ijbiomac.2017.10.183.
89. Kumeta, Y.; Ito, M. Characterization of delta-Guaiene Synthases from Cultured Cells of Aquilaria, Responsible for the Formation of the Sesquiterpenes in Agarwood. *Plant Physiology* **2010**, *154*, 1998-2007, doi:10.1104/pp.110.161828.
90. Kumeta, Y.; Ito, M. Characterization of alpha-humulene synthases responsible for the production of

sesquiterpenes induced by methyl jasmonate in Aquilaria cell culture. *Journal of Natural Medicines* **2016**, *70*, 452-459, doi:10.1007/s11418-016-0999-8.
91. Martin, D.M.; Toub, O.; Chiang, A.; Lo, B.C.; Ohse, S.; Lund, S.T.; Bohlmann, J. The bouquet of grapevine (Vitis vinifera L. cv. Cabernet Sauvignon) flowers arises from the biosynthesis of sesquiterpene volatiles in pollen grains. *P Natl Acad Sci USA* **2009**, *106*, 7245-7250, doi:10.1073/pnas.0901387106.
92. Lucker, J.; Bowen, P.; Bohlmann, J. Vitis vinifera terpenoid cyclases: functional identification of two sesquiterpene synthase cDNAs encoding (+)-valencene synthase and (-)-germacrene D synthase and expression of mono- and sesquiterpene synthases in grapevine flowers and berries. *Phytochemistry* **2004**, *65*, 2649-2659, doi:10.1016/j.phytochem.2004.08.017.
93. Martin, D.M.; Aubourg, S.; Schouwey, M.B.; Daviet, L.; Schalk, M.; Toub, O.; Lund, S.T.; Bohlmann, J. Functional Annotation, Genome Organization and Phylogeny of the Grapevine (Vitis vinifera) Terpene Synthase Gene Family Based on Genome Assembly, FLcDNA Cloning, and Enzyme Assays. *Bmc Plant Biology* **2010**, *10*, doi:10.1186/1471-2229-10-226.
94. Dueholm, B.; Drew, D.P.; Sweetman, C.; Simonsen, H.T. In planta and in silico characterization of five sesquiterpene synthases from Vitis vinifera (cv. Shiraz) berries. *Planta* **2019**, *249*, 59-70, doi:10.1007/s00425-018-2986-7.
95. Falara, V.; Fotopoulos, V.; Margaritis, T.; Anastasaki, T.; Pateraki, I.; Bosabalidis, A.M.; Kafetzopoulos, D.; Demetzos, C.; Pichersky, E.; Kanellis, A.K. Transcriptome analysis approaches for the isolation of trichome-specific genes from the medicinal plant Cistus creticus subsp creticus. *Plant Molecular Biology* **2008**, *68*, 633-651, doi:10.1007/s11103-008-9399-0.
96. Yang, C.-Q.; Wu, X.-M.; Ruan, J.-X.; Hu, W.-L.; Mao, Y.-B.; Chen, X.-Y.; Wang, L.-J. Isolation and characterization of terpene synthases in cotton (Gossypium hirsutum). *Phytochemistry* **2013**, *96*, 46-56, doi:10.1016/j.phytochem.2013.09.009.
97. Chen, X.Y.; Chen, Y.; Heinstein, P.; Davisson, V.J. Cloning, expression, and characterization of (+)-delta-cadinene synthase: a catalyst for cotton phytoalexin biosynthesis. *Archives of biochemistry and biophysics* **1995**, *324*, 255-266, doi:10.1006/abbi.1995.0038.
98. Chen, X.Y.; Wang, M.; Chen, Y.; Davisson, V.J.; Heinstein, P. Cloning and heterologous expression of a second (+)-delta-cadinene synthase from Gossypium arboreum. *Journal of natural products* **1996**, *59*, 944-951, doi:10.1021/np960344w.
99. Srivastava, P.L.; Daramwar, P.P.; Krithika, R.; Pandreka, A.; Shankar, S.S.; Thulasiram, H.V. Functional Characterization of Novel Sesquiterpene Synthases from Indian Sandalwood, Santalum album. *Scientific Reports* **2015**, *5*, doi:10.1038/srep10095.
100. Jones, C.G.; Keeling, C.I.; Ghisalberti, E.L.; Barbour, E.L.; Plummer, J.A.; Bohlmann, J. Isolation of cDNAs and functional characterisation of two multi-product terpene synthase enzymes from sandalwood, Santalum album L. *Archives of Biochemistry and Biophysics* **2008**, *477*, 121-130, doi:10.1016/j.abb.2008.05.008.
101. Zhang, X.; Niu, M.; da Silva, J.A.T.; Zhang, Y.; Yuan, Y.; Jia, Y.; Xiao, Y.; Li, Y.; Fang, L.; Zeng, S.; et al. Identification and functional characterization of three new terpene synthase genes involved in chemical defense and abiotic stresses in Santalum album. *Bmc Plant Biology* **2019**, *19*, doi:10.1186/s12870-019-1720-3.
102. Moniodis, J.; Jones, C.G.; Barbour, E.L.; Plummer, J.A.; Ghisalberti, E.L.; Bohlmann, J. The transcriptome of sesquiterpenoid biosynthesis in heartwood xylem of Western Australian sandalwood (Santalum spicatum). *Phytochemistry* **2015**, *113*, 79-86, doi:10.1016/j.phytochem.2014.12.009.
103. Jones, C.G.; Moniodis, J.; Zulak, K.G.; Scaffidi, A.; Plummer, J.A.; Ghisalberti, E.L.; Barbour, E.L.; Bohlmann, J. Sandalwood Fragrance Biosynthesis Involves Sesquiterpene Synthases of Both the Terpene Synthase (TPS)-a and TPS-b Subfamilies, including Santalene Synthases. *Journal of Biological Chemistry* **2011**, *286*, 17445-17454, doi:10.1074/jbc.M111.231787.
104. Ker, D.-S.; Pang, S.L.; Othman, N.F.; Kumaran, S.; Tan, E.F.; Krishnan, T.; Chan, K.G.; Othman, R.; Hassan, M.; Ng, C.L. Purification and biochemical characterization of recombinant Persicaria minor beta-sesquiphellandrene synthase. *Peerj* **2017**, *5*, doi:10.7717/peerj.2961.
105. Rusdi, N.A.; Goh, H.-H.; Sabri, S.; Ramzi, A.B.; Noor, N.M.; Baharum, S.N. Functional Characterisation of New Sesquiterpene Synthase from the Malaysian Herbal Plant, Polygonum Minus. *Molecules* **2018**, *23*, doi:10.3390/molecules23061370.
106. Henquet, M.G.L.; Prota, N.; van der Hooft, J.J.J.; Varbanova-Herde, M.; Hulzink, R.J.M.; de Vos, M.; Prins, M.; de Both, M.T.J.; Franssen, M.C.R.; Bouwmeester, H.; et al. Identification of a drimenol synthase and drimenol oxidase from Persicaria hydropiper, involved in the biosynthesis of insect deterrent drimanes. *Plant Journal* **2017**, *90*, 1052-1063, doi:10.1111/tpj.13527.
107. Zhou, Y.; Zeng, L.; Liu, X.; Gui, J.; Mei, X.; Fu, X.; Dong, F.; Tang, J.; Zhang, L.; Yang, Z. Formation of (E)-

nerolidol in tea (Camellia sinensis) leaves exposed to multiple stresses during tea manufacturing. *Food Chemistry* **2017**, *231*, 78-86, doi:10.1016/j.foodchem.2017.03.122.
108. Liu, G.-F.; Liu, J.-J.; He, Z.-R.; Wang, F.-M.; Yang, H.; Yan, Y.-F.; Gao, M.-J.; Gruber, M.Y.; Wan, X.-C.; Wei, S. Implementation of CsLIS/NES in linalool biosynthesis involves transcript splicing regulation in Camellia sinensis. *Plant Cell and Environment* **2018**, *41*, 176-186, doi:10.1111/pce.13080.
109. Wang, X.; Zeng, L.; Liao, Y.; Li, J.; Tang, J.; Yang, Z. Formation of alpha-Farnesene in Tea (Camellia sinensis) Leaves Induced by Herbivore-Derived Wounding and Its Effect on Neighboring Tea Plants. *International Journal of Molecular Sciences* **2019**, *20*, doi:10.3390/ijms20174151.
110. Hattan, J.-i.; Shindo, K.; Ito, T.; Shibuya, Y.; Watanabe, A.; Tagaki, C.; Ohno, F.; Sasaki, T.; Ishii, J.; Kondo, A.; et al. Identification of a novel hedycaryol synthase gene isolated from Camellia brevistyla flowers and floral scent of Camellia cultivars. *Planta* **2016**, *243*, 959-972, doi:10.1007/s00425-015-2454-6.
111. Nieuwenhuizen, N.J.; Wang, M.Y.; Matich, A.J.; Green, S.A.; Chen, X.; Yauk, Y.-K.; Beuning, L.L.; Nagegowda, D.A.; Dudareva, N.; Atkinson, R.G. Two terpene synthases are responsible for the major sesquiterpenes emitted from the flowers of kiwifruit (Actinidia deliciosa). *Journal of Experimental Botany* **2009**, *60*, 3203-3219, doi:10.1093/jxb/erp162.
112. Green, S.A.; Chen, X.; Nieuwenhuizen, N.J.; Matich, A.J.; Wang, M.Y.; Bunn, B.J.; Yauk, Y.-K.; Atkinson, R.G. Identification, functional characterization, and regulation of the enzyme responsible for floral (E)-nerolidol biosynthesis in kiwifruit (Actinidia chinensis). *Journal of Experimental Botany* **2012**, *63*, 1951-1967, doi:10.1093/jxb/err393.
113. Iijima, Y.; Davidovich-Rikanati, R.; Fridman, E.; Gang, D.R.; Bar, E.; Lewinsohn, E.; Pichersky, E. The biochemical and molecular basis for the divergent patterns in the biosynthesis of terpenes and phenylpropenes in the peltate glands of three cultivars of basil. *Plant Physiology* **2004**, *136*, 3724-3736, doi:10.1104/pp.104.051318.
114. Landmann, C.; Fink, B.; Festner, M.; Dregus, M.; Engel, K.-H.; Schwab, W. Cloning and functional characterization of three terpene synthases from lavender (Lavandula angustifolia). *Archives of Biochemistry and Biophysics* **2007**, *465*, 417-429, doi:10.1016/j.abb.2007.06.011.
115. Jullien, F.; Moja, S.; Bony, A.; Legrand, S.; Petit, C.; Benabdelkader, T.; Poirot, K.; Fiorucci, S.; Guitton, Y.; Nicole, F.; et al. Isolation and functional characterization of a tau-cadinol synthase, a new sesquiterpene synthase from Lavandula angustifolia. *Plant Molecular Biology* **2014**, *84*, 227-241, doi:10.1007/s11103-013-0131-3.
116. Sarker, L.S.; Demissie, Z.A.; Mahmoud, S.S. Cloning of a sesquiterpene synthase from Lavandula x intermedia glandular trichomes. *Planta* **2013**, *238*, 983-989, doi:10.1007/s00425-013-1937-6.
117. Benabdelkader, T.; Guitton, Y.; Pasquier, B.; Magnard, J.L.; Jullien, F.; Kameli, A.; Legendre, L. Functional characterization of terpene synthases and chemotypic variation in three lavender species of section Stoechas. *Physiologia Plantarum* **2015**, *153*, 43-57, doi:10.1111/ppl.12241.
118. Luo, F.; Ling, Y.; Li, D.-S.; Tang, T.; Liu, Y.-C.; Liu, Y.; Li, S.-H. Characterization of a sesquiterpene cyclase from the glandular trichomes of Leucosceptrum canum for sole production of cedrol in Escherichia coli and Nicotiana benthamiana. *Phytochemistry* **2019**, *162*, 121-128, doi:10.1016/j.phytochem.2019.03.009.
119. Crocoll, C.; Asbach, J.; Novak, J.; Gershenzon, J.; Degenhardt, J. Terpene synthases of oregano (Origanum vulgare L.) and their roles in the pathway and regulation of terpene biosynthesis. *Plant Molecular Biology* **2010**, *73*, 587-603, doi:10.1007/s11103-010-9636-1.
120. Crock, J.; Wildung, M.; Croteau, R. Isolation and bacterial expression of a sesquiterpene synthase cDNA clone from peppermint (Mentha x piperita, L.) that produces the aphid alarm pheromone (E)-beta-farnesene. *P Natl Acad Sci USA* **1997**, *94*, 12833-12838, doi:10.1073/pnas.94.24.12833.
121. Prosser, I.M.; Adams, R.J.; Beale, M.H.; Hawkins, N.D.; Phillips, A.L.; Pickett, J.A.; Field, L.M. Cloning and functional characterisation of a cis-muuroladiene synthase from black peppermint (Mentha x piperita) and direct evidence for a chemotype unable to synthesise farnesene. *Phytochemistry* **2006**, *67*, 1564-1571, doi:10.1016/j.phytochem.2005.06.012.
122. Deguerry, F.; Pastore, L.; Wu, S.; Clark, A.; Chappell, J.; Schalk, M. The diverse sesquiterpene profile of patchouli, Pogostemon cablin, is correlated with a limited number of sesquiterpene synthases. *Archives of Biochemistry and Biophysics* **2006**, *454*, 123-136, doi:10.1016/j.abb.2006.08.006.
123. Hartwig, S.; Frister, T.; Alemdar, S.; Li, Z.; Krings, U.; Berger, R.G.; Scheper, T.; Beutel, S. Expression, purification and activity assay of a patchoulol synthase cDNA variant fused to thioredoxin in Escherichia coli. *Protein Expression and Purification* **2014**, *97*, 61-71, doi:10.1016/j.pep.2014.02.003.
124. Zeng, X.; Liu, C.; Zheng, R.; Cai, X.; Luo, J.; Zou, J.; Wang, C. Emission and Accumulation of Monoterpene and the Key Terpene Synthase (TPS) Associated with Monoterpene Biosynthesis in Osmanthus fragrans Lour. *Frontiers in Plant Science* **2016**, *6*, doi:10.3389/fpls.2015.01232.

125. Nagegowda, D.A.; Gutensohn, M.; Wilkerson, C.G.; Dudareva, N. Two nearly identical terpene synthases catalyze the formation of nerolidol and linalool in snapdragon flowers. *Plant Journal* **2008**, *55*, 224-239, doi:10.1111/j.1365-313X.2008.03496.x.
126. Attia, M.; Kim, S.-U.; Ro, D.-K. Molecular cloning and characterization of (+)-epi-alpha-bisabolol synthase, catalyzing the first step in the biosynthesis of the natural sweetener, hernandulcin, in Lippia dulcis. *Archives of Biochemistry and Biophysics* **2012**, *527*, 37-44, doi:10.1016/j.abb.2012.07.010.
127. Sallaud, C.; Rontein, D.; Onillon, S.; Jabes, F.; Duffe, P.; Giacalone, C.; Thoraval, S.; Escoffier, C.; Herbette, G.; Leonhardt, N.; et al. A Novel Pathway for Sesquiterpene Biosynthesis from Z,Z-Farnesyl Pyrophosphate in the Wild Tomato Solanum habrochaites. *Plant Cell* **2009**, *21*, 301-317, doi:10.1105/tpc.107.057885.
128. Bleeker, P.M.; Spyropoulou, E.A.; Diergaarde, P.J.; Volpin, H.; De Both, M.T.J.; Zerbe, P.; Bohlmann, J.; Falara, V.; Matsuba, Y.; Pichersky, E.; et al. RNA-seq discovery, functional characterization, and comparison of sesquiterpene synthases from Solanum lycopersicum and Solanum habrochaites trichomes. *Plant Molecular Biology* **2011**, *77*, 323-336, doi:10.1007/s11103-011-9813-x.
129. van der Hoeven, R.S.; Monforte, A.J.; Breeden, D.; Tanksley, S.D.; Steffens, J.C. Genetic control and evolution of sesquiterpene biosynthesis in Lycopersicon esculentum and L-hirsutum. *Plant Cell* **2000**, *12*, 2283-2294, doi:10.1105/tpc.12.11.2283.
130. Gonzales-Vigil, E.; Hufnagel, D.E.; Kim, J.; Last, R.L.; Barry, C.S. Evolution of TPS20-related terpene synthases influences chemical diversity in the glandular trichomes of the wild tomato relative Solanum habrochaites. *Plant Journal* **2012**, *71*, 921-935, doi:10.1111/j.1365-313X.2012.05040.x.
131. Falara, V.; Akhtar, T.A.; Nguyen, T.T.H.; Spyropoulou, E.A.; Bleeker, P.M.; Schauvinhold, I.; Matsuba, Y.; Bonini, M.E.; Schilmiller, A.L.; Last, R.L.; et al. The Tomato Terpene Synthase Gene Family. *Plant Physiology* **2011**, *157*, 770-789, doi:10.1104/pp.111.179648.
132. Colby, S.M.; Crock, J.; Dowdle-Rizzo, B.; Lemaux, P.G.; Croteau, R. Germacrene C synthase from Lycopersicon esculentum cv. VFNT Cherry tomato: cDNA isolation, characterization, and bacterial expression of the multiple product sesquiterpene cyclase. *P Natl Acad Sci USA* **1998**, *95*, 2216-2221, doi:10.1073/pnas.95.5.2216.
133. Tsaballa, A.; Nikolaidis, A.; Trikka, F.; Ignea, C.; Kampranis, S.C.; Makris, A.M.; Argiriou, A. Use of the de novo transcriptome analysis of silver-leaf nightshade (Solanum elaeagnifolium) to identify gene expression changes associated with wounding and terpene biosynthesis. *Bmc Genomics* **2015**, *16*, doi:10.1186/s12864-015-1738-3.
134. Back, K.; Chappell, J. Cloning and bacterial expression of a sesquiterpene cyclase from Hyoscyamus muticus and its molecular comparison to related terpene cyclases. *The Journal of biological chemistry* **1995**, *270*, 7375-7381, doi:10.1074/jbc.270.13.7375.
135. Back, K.W.; He, S.L.; Kim, K.U.; Shin, D.H. Cloning and bacterial expression of sesquiterpene cyclase, a key branch point enzyme for the synthesis of sesquiterpenoid phytoalexin capsidiol in UV-challenged leaves of Capsicum annuum. *Plant and Cell Physiology* **1998**, *39*, 899-904, doi:10.1093/oxfordjournals.pcp.a029452.
136. Wu, S.Q.; Schoenbeck, M.A.; Greenhagen, B.T.; Takahashi, S.; Lee, S.B.; Coates, R.M.; Chappell, J. Surrogate splicing for functional analysis of sesquiterpene synthase genes. *Plant Physiology* **2005**, *138*, 1322-1333, doi:10.1104/pp.105.059386.
137. Back, K.; Yin, S.; Chappell, J. Expression of a plant sesquiterpene cyclase gene in Escherichia coli. *Archives of biochemistry and biophysics* **1994**, *315*, 527-532, doi:10.1006/abbi.1994.1533.
138. Bohlmann, J.; Stauber, E.J.; Krock, B.; Oldham, N.J.; Gershenzon, J.; Baldwin, I.T. Gene expression of 5-epi-aristolochene synthase and formation of capsidiol in roots of Nicotiana attenuata and N-sylvestris. *Phytochemistry* **2002**, *60*, 109-116, doi:10.1016/s0031-9422(02)00080-8.
139. Son, Y.-J.; Kwon, M.; Ro, D.-K.; Kim, S.-U. Enantioselective microbial synthesis of the indigenous natural product (-)-alpha-bisabolol by a sesquiterpene synthase from chamomile (Matricaria recutita). *Biochemical Journal* **2014**, *463*, 239-248, doi:10.1042/bj20140306.
140. Irmisch, S.; Krause, S.T.; Kunert, G.; Gershenzon, J.; Degenhardt, J.; Koellner, T.G. The organ-specific expression of terpene synthase genes contributes to the terpene hydrocarbon composition of chamomile essential oils. *Bmc Plant Biology* **2012**, *12*, doi:10.1186/1471-2229-12-84.
141. Su, S.; Liu, X.; Pan, G.; Hou, X.; Zhang, H.; Yuan, Y. In vitro characterization of a (E)-beta-farnesene synthase from Matricaria recutita L. and its up-regulation by methyl jasmonate. *Gene* **2015**, *571*, 58-64, doi:10.1016/j.gene.2015.06.037.
142. Prosser, I.; Phillips, A.L.; Gittings, S.; Lewis, M.J.; Hooper, A.M.; Pickett, J.A.; Beale, M.H. (+)-(10R)-germacrene A synthase from goldenrod, Solidago canadensis; cDNA isolation, bacterial expression and

functional analysis. *Phytochemistry* **2002**, *60*, 691-702, doi:10.1016/s0031-9422(02)00165-6.
143. Prosser, I.; Altug, I.G.; Phillips, A.L.; Konig, W.A.; Bouwmeester, H.J.; Beale, M.H. Enantiospecific (+)- and (-)-germacrene D synthases, cloned from goldenrod, reveal a functionally active variant of the universal isoprenoid-biosynthesis aspartate-rich motif. *Archives of Biochemistry and Biophysics* **2004**, *432*, 136-144, doi:10.1016/j.abb.2004.06.030.
144. Kim, M.Y.; Chang, Y.J.; Bang, M.H.; Baek, N.I.; Jin, J.; Lee, C.H.; Kim, S.U. cDNA isolation and characterization of (+)-germacrene A synthase from Ixeris dentata form. albiflora Hara. *Journal of Plant Biology* **2005**, *48*, 178-186, doi:10.1007/bf03030406.
145. Trinh-Don, N.; Faraldos, J.A.; Vardakou, M.; Salmon, M.; O'Maille, P.E.; Ro, D.-K. Discovery of germacrene A synthases in Barnadesia spinosa: The first committed step in sesquiterpene lactone biosynthesis in the basal member of the Asteraceae. *Biochemical and Biophysical Research Communications* **2016**, *479*, 622-627, doi:10.1016/j.bbrc.2016.09.165.
146. Huber, M.; Epping, J.; Gronover, C.S.; Fricke, J.; Aziz, Z.; Brillatz, T.; Swyers, M.; Koellner, T.G.; Vogel, H.; Hammerbacher, A.; et al. A Latex Metabolite Benefits Plant Fitness under Root Herbivore Attack. *Plos Biology* **2016**, *14*, doi:10.1371/journal.pbio.1002332.
147. Ramirez, A.M.; Saillard, N.; Yang, T.; Franssen, M.C.R.; Bouwmeester, H.J.; Jongsma, M.A. Biosynthesis of Sesquiterpene Lactones in Pyrethrum (Tanacetum cinerariifolium). *Plos One* **2013**, *8*, doi:10.1371/journal.pone.0065030.
148. Majdi, M.; Liu, Q.; Karimzadeh, G.; Malboobi, M.A.; Beekwilder, J.; Cankar, K.; de Vos, R.; Todorovic, S.; Simonovic, A.; Bouwmeester, H. Biosynthesis and localization of parthenolide in glandular trichomes of feverfew (Tanacetum parthenium L. Schulz Bip.). *Phytochemistry* **2011**, *72*, 1739-1750, doi:10.1016/j.phytochem.2011.04.021.
149. Alves Gomes Albertti, L.; Delatte, T.L.; Souza de Farias, K.; Galdi Boaretto, A.; Verstappen, F.; van Houwelingen, A.; Cankar, K.; Carollo, C.A.; Bouwmeester, H.J.; Beekwilder, J. Identification of the Bisabolol Synthase in the Endangered Candeia Tree (Eremanthus erythropappus (DC) McLeisch). *Frontiers in plant science* **2018**, *9*, 1340-1340, doi:10.3389/fpls.2018.01340.
150. Bouwmeester, H.J.; Kodde, J.; Verstappen, F.W.A.; Altug, I.G.; de Kraker, J.W.; Wallaart, T.E. Isolation and characterization of two germacrene A synthase cDNA clones from chicory. *Plant Physiology* **2002**, *129*, 134-144, doi:10.1104/pp.001024.
151. Bennett, M.H.; Mansfield, J.W.; Lewis, M.J.; Beale, M.H. Cloning and expression of sesquiterpene synthase genes from lettuce (Lactuca sativa L.). *Phytochemistry* **2002**, *60*, 255-261, doi:10.1016/s0031-9422(02)00103-6.
152. Aschenbrenner, A.-K.; Kwon, M.; Conrad, J.; Ro, D.-K.; Spring, O. Identification and characterization of two bisabolene synthases from linear glandular trichomes of sunflower (Helianthus annuus L., Asteraceae). *Phytochemistry* **2016**, *124*, 29-37, doi:10.1016/j.phytochem.2016.01.009.
153. Goepfert, J.C.; MacNevin, G.; Ro, D.-K.; Spring, O. Identification, functional characterization and developmental regulation of sesquiterpene synthases from sunflower capitate glandular trichomes. *Bmc Plant Biology* **2009**, *9*, doi:10.1186/1471-2229-9-86.
154. Goepfert, J.; Buelow, A.-K.; Spring, O. Identification and Functional Characterization of a new Sunflower Germacrene A Synthase (HaGAS3). *Natural Product Communications* **2010**, *5*, 709-715.
155. Li, Y.; Chen, F.; Li, Z.; Li, C.; Zhang, Y. Identification and Functional Characterization of Sesquiterpene Synthases from Xanthium strumarium. *Plant and Cell Physiology* **2016**, *57*, 630-641, doi:10.1093/pcp/pcw019.
156. Gfeller, V.; Huber, M.; Foerster, C.; Huang, W.; Koellner, T.G.; Erb, M. Root volatiles in plant-plant interactions I: High root sesquiterpene release is associated with increased germination and growth of plant neighbours. *Plant Cell and Environment* **2019**, *42*, 1950-1963, doi:10.1111/pce.13532.
157. Pazouki, L.; Memari, H.R.; Kaennaste, A.; Bichele, R.; Niinemets, U. Germacrene A synthase in yarrow (Achillea millefolium) is an enzyme with mixed substrate specificity: gene cloning, functional characterization and expression analysis. *Frontiers in Plant Science* **2015**, *6*, doi:10.3389/fpls.2015.00111.
158. Shen, Q.-Q.; Wang, L.-P.; Liang, J.; Liu, L.-J.; Wang, Q. Functional characterization of SsNES responsible for nerolidol biosynthesis in Senecio scandens. *China journal of Chinese materia medica* **2019**, *44*, 1334-1340, doi:10.19540/j.cnki.cjcmm.20181204.009.
159. Menin, B.; Comino, C.; Portis, E.; Moglia, A.; Cankar, K.; Bouwmeester, H.J.; Lanteri, S.; Beekwilder, J. Genetic mapping and characterization of the globe artichoke (+)-germacrene A synthase gene, encoding the first dedicated enzyme for biosynthesis of the bitter sesquiterpene lactone cynaropicrin. *Plant Science* **2012**, *190*, 1-8, doi:10.1016/j.plantsci.2012.03.006.
160. Yahyaa, M.; Tholl, D.; Cormier, G.; Jensen, R.; Simon, P.W.; Ibdah, M. Identification and Characterization

of Terpene Synthases Potentially Involved in the Formation of Volatile Terpenes in Carrot (Daucus carota L.) Roots. *J Agr Food Chem* **2015**, *63*, 4870-4878, doi:10.1021/acs.jafc.5b00546.
161. Muangphrom, P.; Seki, H.; Suzuki, M.; Komori, A.; Nishiwaki, M.; Mikawa, R.; Fukushima, E.O.; Muranaka, T. Functional Analysis of Amorpha-4,11-Diene Synthase (ADS) Homologs from Non-Artemisinin-Producing Artemisia Species: The Discovery of Novel Koidzumiol and (+)-alpha-Bisabolol Synthases. *Plant and Cell Physiology* **2016**, *57*, 1678-1688, doi:10.1093/pcp/pcw094.
162. Muangphrom, P.; Seki, H.; Matsumoto, S.; Nishiwaki, M.; Fukushima, E.O.; Muranaka, T. Identification and characterization of a novel sesquiterpene synthase, 4-amorphen-11-ol synthase, from Artemisia maritima. *Plant Biotechnology* **2018**, *35*, 113-121, doi:10.5511/plantbiotechnology.18.0324a.
163. Li, J.-X.; Fang, X.; Zhao, Q.; Ruan, J.-X.; Yang, C.-Q.; Wang, L.-J.; Miller, D.J.; Faraldos, J.A.; Allemann, R.K.; Chen, X.-Y.; et al. Rational engineering of plasticity residues of sesquiterpene synthases from Artemisia annua: product specificity and catalytic efficiency. *Biochemical Journal* **2013**, *451*, 417-426, doi:10.1042/bj20130041.
164. Bertea, C.M.; Voster, A.; Verstappen, F.W.A.; Maffei, M.; Beekwilder, J.; Bouwmeester, H.J. Isoprenoid biosynthesis in Artemisia annua: Cloning and heterologous expression of a germacrene A synthase from a glandular trichome cDNA library. *Archives of Biochemistry and Biophysics* **2006**, *448*, 3-12, doi:10.1016/j.abb.2006.02.026.
165. Cai, Y.; Jia, J.W.; Crock, J.; Lin, Z.X.; Chen, X.Y.; Croteau, R. A cDNA clone for beta-caryophyllene synthase from Artemisia annua. *Phytochemistry* **2002**, *61*, 523-529, doi:10.1016/s0031-9422(02)00265-0.
166. Mercke, P.; Bengtsson, M.; Bouwmeester, H.J.; Posthumus, M.A.; Brodelius, P.E. Molecular cloning, expression, and characterization of amorpha-4,11-diene synthase, a key enzyme of artemisinin biosynthesis in Artemisia annua L. *Archives of Biochemistry and Biophysics* **2000**, *381*, 173-180, doi:10.1006/abbi.2000.1962.
167. Mercke, P.; Crock, J.; Croteau, R.; Brodelius, P.E. Cloning, expression, and characterization of epi-cedrol synthase, a sesquiterpene cyclase from Artemisia annua L. *Archives of Biochemistry and Biophysics* **1999**, *369*, 213-222, doi:10.1006/abbi.1999.1358.
168. Picaud, S.; Brodelius, M.; Brodelius, P.E. Expression, purification and characterization of recombinant (E)-beta-farnesene synthase from Artemisia annua. *Phytochemistry* **2005**, *66*, 961-967, doi:10.1016/j.phytochem.2005.03.027.
169. Muangphrom, P.; Misaki, M.; Suzuki, M.; Shimomura, M.; Suzuki, H.; Seki, H.; Muranaka, T. Identification and characterization of (+)-alpha-bisabolol and 7-epi-silphiperfol-5-ene synthases from Artemisia abrotanum. *Phytochemistry* **2019**, *164*, 144-153, doi:10.1016/j.phytochem.2019.05.010.
170. Pickel, B.; Drew, D.P.; Manczak, T.; Weitzel, C.; Simonsen, H.T.; Ro, D.-K. Identification and characterization of a kunzeaol synthase from Thapsia garganica: implications for the biosynthesis of the pharmaceutical thapsigargin. *Biochemical Journal* **2012**, *448*, 261-271, doi:10.1042/bj20120654.
171. Andersen, T.B.; Rasmussen, S.A.; Christensen, S.B.; Simonsen, H.T. Biosynthesis of tovarol and other sesquiterpenoids in Thapsia laciniata Rouy. *Phytochemistry* **2019**, *157*, 168-174, doi:10.1016/j.phytochem.2018.10.027.
172. Trine, A.; Federico, C.; Henrik, S. Optimization of Biochemical Screening Methods for Volatile and Unstable Sesquiterpenoids Using HS-SPME-GC-MS. *Chromatography* **2015**, *2*, 277-292.
173. Pyle, B.W.; Tran, H.T.; Pickel, B.; Haslam, T.M.; Gao, Z.; MacNevin, G.; Vederas, J.C.; Kim, S.-U.; Ro, D.-K. Enzymatic synthesis of valerena-4,7(11)-diene by a unique sesquiterpene synthase from the valerian plant (Valeriana officinalis). *Febs Journal* **2012**, *279*, 3136-3146, doi:10.1111/j.1742-4658.2012.08692.x.
174. Yeo, Y.-S.; Nybo, S.E.; Chittiboyina, A.G.; Weerasooriya, A.D.; Wang, Y.-H.; Gongora-Castillo, E.; Vaillancourt, B.; Buell, C.R.; DellaPenna, D.; Celiz, M.D.; et al. Functional Identification of Valerena-1,10-diene Synthase, a Terpene Synthase Catalyzing a Unique Chemical Cascade in the Biosynthesis of Biologically Active Sesquiterpenes in Valeriana officinalis. *Journal of Biological Chemistry* **2013**, *288*, 3163-3173, doi:10.1074/jbc.M112.415836.
175. Kwon, M.; Cochrane, S.A.; Vederas, J.C.; Ro, D.-K. Molecular cloning and characterization of drimenol synthase from valerian plant (*Valeriana officinalis*). *Febs Letters* **2014**, *588*, 4597-4603, doi:10.1016/j.febslet.2014.10.031.
